# Discovery, Bioactivity Evaluation, Biosynthetic Gene Cluster Identification, and Heterologous Expression of Novel Albofungin Derivatives

**DOI:** 10.3389/fmicb.2021.635268

**Published:** 2021-02-01

**Authors:** Weiyi She, Wenkang Ye, Aifang Cheng, Xin Liu, Jianwei Tang, Yi Lan, Feng Chen, Pei-Yuan Qian

**Affiliations:** ^1^SZU-HKUST Joint Ph.D. Program in Marine Environmental Science, Shenzhen University, Shenzhen, China; ^2^Hong Kong Branch of the Southern Marine Science and Engineering Guangdong, Laboratory (Guangzhou), Hong Kong University of Science and Technology, Hong Kong, China; ^3^Division of Ocean Science, Hong Kong University of Science and Technology, Hong Kong, China; ^4^Institute for Advanced Study, Shenzhen University, Shenzhen, China

**Keywords:** polycyclic xanthones, actinobacteria, heterologous expression, antibiotics, antitumor, albofungin biosynthetic gene cluster, genome mining

## Abstract

The crude extract of *Streptomyces chrestomyceticus* exhibited strong and broad activities against most “ESKAPE pathogens.” We conducted a comprehensive chemical investigation for secondary metabolites from the *S. chrestomyceticus* strain and identified two novel albofungin (*alb*) derivatives, i.e., albofungins A (1) and B (2), along with two known compounds, i.e., albofungin (3) and chloroalbofungin (4). The chemical structures of the novel compounds were elucidated using HRMS, 1D and 2D NMR, and electronic circular dichroism spectroscopy. The draft genome of *S. chrestomyceticus* was sequenced, and a 72 kb albofungin (*alb*) gene cluster with 72 open reading frames encoding type II polyketide synthases (PKSs), regulators, and transporters, and tailoring enzymes were identified using bioinformatics analysis. The *alb* gene cluster was confirmed using the heterologous expression in *Streptomyces coelicolor*, which successfully produced the compounds 3 and 4. Furthermore, compounds 1–4 displayed remarkable activities against Gram-positive bacteria and antitumor activities toward various cancer cells. Notably, compounds 1 and 3 showed potent activities against Gram-negative pathogenic bacteria. The terminal deoxynucleotidyl transferase (dUTP) nick-end labeling and flow cytometry analysis verified that compound 1 inhibited cancer cell proliferation by inducing cellular apoptosis. These results indicated that albofungins might be potential candidates for the development of antibiotics and antitumor drugs.

## Introduction

The rise of multidrug-resistant pathogen infections brings a considerable burden on public health and generates high economic costs globally. Among these infections, the nosocomial infection is usually caused by a group of “ESKAPE pathogens,” comprising Gram-positive and Gram-negative bacteria, including *Enterococcus faecium*, *Staphylococcus aureus*, *Klebsiella pneumoniae*, *Acinetobacter baumannii*, *Pseudomonas aeruginosa*, and *Enterobacter* species (Rice, 2008). These ESKAPE pathogens carry antimicrobial resistance genes and prevalently exist in the hospital environment (Santajit and Indrawattana, 2016). Also, the widespread and inappropriate use of antibiotics leads to antimicrobial resistance, which becomes a severe public health problem. Therefore, new antibacterial agents targeting the “superbugs” should be developed urgently (Prestinaci et al., 2015).

We mined the actinomycetes, which are Gram-positive bacteria generating many bioactive secondary metabolites with chemical diversity and novel structures originating from marine and soil environments, to find novel antibiotics (Genilloud, 2017). *Streptomyces*, a genus of actinomycetes, is one of the most important sources of new antibiotics for decades, producing over two-thirds of natural antibiotics (e.g., neomycin, kanamycin, vancomycin, and rifamycin), which successfully entered the market and are applied in clinical therapies (de Lima Procópio et al., 2012). Albofungin, which was isolated from *Actinomyces* species, belongs to the polycyclic xanthone polyketide family, featuring a highly oxygenated hexacyclic xanthone ring (Fukushima et al., 1973). This family of compounds usually exhibits diverse biological activities, such as antibiotics, antifungal, anthelmintic, and potent antitumor activity (Masters and Bräse, 2012; Winter et al., 2013). However, the antitumor mechanism behind albofungin has not been investigated. Apoptosis, is regarded as an important part of various processes of a biological organism, including normal cell renewal, development of the immune system, and chemical-induced cell death. Apoptosis is a form of programmed cell death and different from necrosis, wherein cells die due to injury. During the apoptosis process, normal cells undergo chromatin condensation, nuclear fragmentation, and blending, and form an apoptosis body (Elmore, 2007).

The antiSMASH provides a comprehensive analysis of the microbial genomes for revealing various secondary metabolites encoded by their biosynthetic gene clusters before recognizing their structures (Medema et al., 2011; Blin et al., 2019). The biosynthesis of type II polyketides usually starts with loading the acetate onto the acyl carrier protein and transferring to the active sites of the ketone synthase. Afterward, malonyl-CoA is used as an extender unit to provide extended polyketide chains. The long polyketide chain is subsequently maintained through cyclization by cyclase, reduction by keto-reductase, and aromatization by aromatase to form the backbone of the aromatic polyketide (Zhang et al., 2017). Various tailoring enzymes, such as oxygenase, reductase, methyltransferase, halogenase, and glycosyltransferase enzymes, participate in the modifications of the polyketide backbone to generate diverse aromatic polyketides (Shen, 2003; Risdian et al., 2019).

The diverse bioactivities and unique structures of polycyclic xanthones attracted researchers to further investigate their biosynthesis. The biosynthetic gene clusters of antibiotics FD-594, lysolipin, and xantholipin from *Streptomyces* spp. were cloned and sequenced, and bioinformatics analysis revealed their putative functions of the open reading frames (Lopez et al., 2010; Kudo et al., 2011). The mutagenesis and metabolite analysis of five pathway-blocked mutants further identified the biosynthesis pathway of xantholipin (Zhang et al., 2012). In our study, the crude extract of *Streptomyces chrestomyceticus* shows strong bioactivities against “ESKAPE” Gram-positive and Gram-negative bacteria ranging from the micromolar concentration to the nanomolar concentration. The genome-based antiSMASH analysis reveals a gene cluster (*alb*, 72 kb) in *S. chrestomyceticus* for the biosynthesis of type II polyketide polycyclic xanthones. Furthermore, we have conducted a chemical investigation on the crude extract of *S. chrestomyceticus* and discovered two novel albofungin derivatives [albofungins A (1) and B (2)] together with two known compounds, namely, albofungin (3) and chloroalbofungin (4). Herein, we have reported the isolation, purification, and structure elucidation for 1 and 2 from *S. chrestomyceticus* and their antibacterial activity. Notably, these compounds also exhibit potent antitumor activity. Further, cell death mechanism study reveals that compound 1 inhibits cancer cell proliferation by inducing apoptosis. Moreover, we have successfully conducted a heterologous expression for the whole albofungin biosynthetic gene cluster in *Streptomyces coelicolor* and proposed a possible biosynthesis pathway based on the genome analysis of albofungin derivatives.

## Experimental Section

### General Experimental Procedures

Preparative high performance liquid chromatography (HPLC) was performed using the Phenomenex Luna C18 column (100A, 250 × 21.2 mm, 5 sm) by using the Waters 2695 Separations Module, and the eluate was monitored at a UV wavelength of 210 nm (Waters 2998 Photodiode Array Detector; Milford, United States). ^1^H NMR was performed on the 500 and the 800 MHz Varian spectrometers, and ^13^C NMR spectra were obtained on the 200 MHz Varian spectrometers. Standard 2D NMR experimental spectra, including HSQC, heteronuclear multiple bond correlation (HMBC), and COSY, were collected at 25°C. MS data were recorded from the Bruker ultrafleXtreme ultrahigh-resolution TOF LC-MS system. Optical rotations were determined using the Jasco P-2000 Polarimeter. Circular dichroism spectra were obtained using the Chirascan circular dichroism spectrometer.

### Extraction and Isolation of Compounds 1–4

The culture of *S. chrestomyceticus* BCC 24770 (25 L) was extracted using the same volume of ethyl acetate three times and then evaporated under reduced pressure to obtain a dried crude extract (27 g). This crude extract was dissolved in MeOH, loaded into the C18 silica gel column chromatography for fractionation with an increasing gradient of MeOH:H_2_O (20:80–100:0) to obtain nine fractions (Fractions 1–9). All fractions were systematically analyzed using HPLC, and fractions 5–7 with similar UV patterns were further separated. Fraction 5 was loaded to the Sephadex LH-20 column (eluted with MeOH) to generate four subfractions (Fractions 5–1 to 5–4). Fraction 5–4 was subjected to preparative HPLC (45% acetonitrile–55% H_2_O with 0.5‰ trifluoroacetic acid) to yield compound 1 (15 mg). Fraction 6 was further purified with 50% acetonitrile using preparative HPLC to obtain compounds 2 (2 mg) and 3 (2.1 g). Fraction 7 was purified using preparative HPLC (60% acetonitrile with 0.5‰ trifluoroacetic acid) to yield compound 4 (670 mg).

### Analytical Data

Albofungin A (1): yellow powder; [α]^25^_D_-417 (*c* 1.2, MeOH); UV *λ*_max_ (MeOH) nm 227, 252, 300, and 376; ^1^H and ^13^C NMR data, see Table 1; HRMS *m/z* 507.1398 (M + H)^+^ (calculated for C_26_H_23_N_2_O_9_, 507.1359).

**Table 1:** ^1^H and ^13^C NMR Data for 1–3 in DMSO-*d*6.

	1*^a^*		2*^b^*		3*^c^*	
Position	*δ*_C_, type	*δ*_H_ (*J* in Hz)	*δ*_C_, type	*δ*_H_ (*J* in Hz)	δ_C_, type	*δ*_H_ (*J* in Hz)
1	163.3, C		165.9, C		163.3, C	
2	109.2, C		109.3, C		109.2, C	
3	156.8, C		157.6, C		156.8, C	
4	112.9, C		113.2, C		112.8, C	
5	111.4, C		111.8, C		111.6, C	
6	149.7, C		149.6, C		149.6, C	
7	109.8, C		109.9, C		109.8, C	
8	182.3, C		182.1, C		182.1, C	
9	119.6, C		120.4, C		120.3, C	
10	59.0, CH	4.83, t (3.5)	58.8, CH	4.83, m	58.8, CH	4.82, t (3.5)
11	28.2, CH_2_	a 1.72, m b 1.81, m	28.0, CH_2_	a 1.73, m b 1.83, m	27.9, CH_2_	a 1.72, m b 1.81, m
12	26.6, CH_2_	a 1.94, m b 2.08, m	22.8, CH_2_	a 2.06, m b 2.08, m	22.8, CH_2_	2.06, m
13	65.5, CH	4.58, m	74.7, CH	4.44, dd (8.9, 6.6)	74.7, CH	4.40, dd (8.8, 6.8)
14	167.1, C		165.2, C		165.1, C	
16	142.9, C		142.7, C		142.7, C	
17	130.3, C		130.3, C		130.3, C	
18	129.9, C		130.1, C		130.1, C	
19	72.1, CH	4.97, dd (13.1, 4.7)	72.1, CH	4.96, dd (13.1, 4.7)	72.1, CH	4.95, dd (13.1, 4.7)
20	35.9, CH_2_	a 2.76, t (13.6) b 3.23, dd (13.9, 4.7)	35.9, CH_2_	a 2.75, t (13.4) b 3.22, dd (13.9, 4.7)	36.0, CH_2_	a 2.75, t (13.4) b 3.22, dd (13.9, 4.7)
21	140.4, C		140.9, C		140.4, C	
22	113.8, CH	7.01, s	113.6, CH	6.98, s	113.9, CH	7.00, s
23	136.2, C		137.1, C		139.5, C	
24	105.3, CH	6.62, s	106.5, CH	6.62, s	105.3, CH	6.59, s
25	141.6, C		141.3, C		141.7, C	
26	18.9, CH_3_	2.45, s	20.1, CH_3_	2.45, s	19.0, CH_3_	2.44, s
27	90.5, CH_2_	5.42, d (5.9) 5.60, d (5.9)	90.4, CH_2_	a 5.42, d (5.9) b 5.61, d (5.9)	90.7, CH_2_	a 5.40, d (5.9) b 5.60, d (5.9)
NH_2_		5.90, s				
N-CH_3_			30.3, CH_3_	3.54, s		
3-OH		13.58, s		13.97, s		13.57, s
6-OH		13.07, s		12.93, s		12.94, s
10-OH				5.14, d (4.7)		
13-OCH_3_			57.8, CH_3_	3.56, s	57.8, CH_3_	3.55, s

^a1^H and ^13^C data were recorded at 500 and 200 MHz, respectively. ^b1^H and ^13^C data were recorded at 800 and 200 MHz, respectively. ^c1^H and ^13^C data were recorded at 500 and 100 MHz, respectively.

Albofungin B (2): yellow powder; [α]^25^_D_ −126 (*c* 0.5, MeOH); UV *λ*_max_ (MeOH) nm 227, 252, 300, and 376; ^1^H and ^13^C NMR data, see Table 1; HRMS *m/z* 520.1602 (M + H)^+^ (calculated for C_28_H_26_NO_9_, 520.1563).

### X-Ray Crystallography

3 and its chloro-substituted derivative, 4, were crystallized using MeOH under room temperature for about 7 days at a controlled evaporation rate. Yellowish plates were observed at the bottom of the glass tube. Powder diffractograms were obtained on single-crystal powders at room temperature using Cu-Kα radiation on the PANAlytical X’Pert PRO diffractometer with a 1D X’celerator detector or the PANAlytical Aeris benchtop powder X-ray diffractometer. The single-crystal X-ray structures of 3 and 4 were determined at 100 K on the Rigaku Oxford Diffraction Supernova operating with a microfocus Cu-Kα source and the Atlas detector. Their absolute stereochemistries were confirmed using the refined Flack parameter value.

### Genomic DNA Extraction and Putative Gene Cluster for Polycyclic Xanthone

The BCC 24770 was cultured at the GYM agar plate for 7 days at 30°C, and the mycelium was used for the genomic DNA extraction by following the protocol in the TIANamp Bacteria DNA Kit (DP302). The quality and the quantity of DNA were evaluated using the BioDrop μLITE (BioDrop, Cambridge, United Kingdom). A 350 bp inserted-size library was constructed using the qualified DNA. The library was further sequenced on the Illumina NovaSeq 6000 platform to generate paired-end reads with a length of 150 bp. The Trimmomatic version 0.38 (Bolger et al., 2014) was used to trim low-quality reads based on the raw sequencing reads with default settings and remove the adapters with “ILLUMINACLIP: Truseq3-PE-2.fa.” Clean reads were used for assembling the bacterial genome by using the SPAdes version 3.13.0 (Bankevich et al., 2012) with the settings “–careful −k 47,67,87,107,127.” The MaxBin version 2.2.7 (Wu et al., 2015) was further used to remove the potential contamination of the raw assembled contigs *via* genome binning. The assembly quality, including the contamination and the completeness, was assessed using the CheckM version 1.0.13 (Parks et al., 2015). The genomic DNA was analyzed using the antiSMASH^1^ and revealed a gene cluster 8.1 for putative polycyclic xanthone.

### Cell Culture

HeLa, MCF 7, and Hep G2 cells were cultured in Dulbecco’s modified Eagle medium (DMEM) supplemented with 10% fetal bovine serum (FBS) and 1% penicillin and streptomycin at 37°C and 5% CO_2_.

### Cell Viability and TdT-Mediated dUTP Nick End Labeling Assays

The cell viability assay was performed using the MTT assay. Cells were seeded into 96-well plates at a density of 5,000 cells per well, cultured in DMEM with 10% FBS and 1% penicillin and streptomycin, and incubated at 37°C and 5% CO_2_ for 24 h. The compounds with different concentrations were added into the culture medium for another 24 h. The MTT (20 μl, 5 mg/ml) was added into each well and incubated at 37°C for 4 h. The medium was discarded, and formazan was dissolved in 150 μl DMSO. The absorbance was measured using the Multiskan™ FC microplate photometer at 570 nm. The IC_50_ value was analyzed using the GraphPad Prism software.

For the TdT-mediated deoxynucleotidyl transferase (dUTP) nick end labeling (TUNEL) assay, cells were seeded on coverslips at the bottom of 24-well plates, incubated at 37°C and 5% CO_2_ for 24 h, and treated with 0.008, 0.016, and 0.032 μM of compound 1 for another 24 h. Cells were fixed with 4% paraformaldehyde at room temperature for 20 min and permeabilized with 0.1% Triton X-100 dissolved in PBS. The TUNEL assay was conducted following the manufacturer’s instructions (Thermos Fisher Scientific, Foster City, CA, United States). After washing twice with PBS, cells were stained with Hoechst 33342 (Sigma) and mounted on glass slides. Images were captured using the Zeiss Cell Discoverer 7 automated microscope. The stained positive cell ratio was counted and analyzed using the Image J software.

### Flow Cytometry Analysis of Cell Apoptosis

The eBioscience™ Annexin V Apoptosis Detection Kit FITC (Thermo Fisher Scientific, Foster City, CA, United States) was used to detect cell apoptosis in accordance with the manufacturer’s instructions. HeLa and MCF 7 cells were treated with or without compound 1 in 6-well plates, incubated at 37°C for 24 h, and harvested by washing twice with PBS and once with the Annexin V binding buffer. Cells were resuspended in 100 μl Annexin V binding buffer and stained with 5 μl Annexin V and 5 μl PI. For each experiment, 5,000 stained cells were analyzed using flow cytometry (BD FACSAria™ III, BD Biosciences, United States). Each experiment was repeated thrice.

### Antibacterial Activity Assay

The minimal inhibition concentration (MIC) was determined using the broth microdilution in accordance with the CLSI guidelines. Isolated compounds were tested against pathogenic bacteria, including MRSA ATCC 43300, *S. aureus* ATCC 25923, *S. aureus* B04, *B. subtilis* zk31, *K. pneumoniae* NRRL-B-3521, *K. pneumoniae* NRRL-B-408, *A. baumannii* B-65371, *Enterobacter cloacae* NRRL-B-425, *Escherichia coli* k12, and *E. coli* MG1655. The overnight culture was briefly diluted into 1 × 10^5^ CFU ml^−1^ in Mueller Hinton broth and added with different concentrations of compounds in 96-well plates. Plates were incubated at 37°C for 24 h. The lowest concentration of the compound with no viable growth was regarded as the MIC value.

### Bacterial Artificial Chromosome Library Construction and Screening

*Streptomyces chrestomyceticus* BCC 24770 was cultivated in 0.4% glucose, 0.4% yeast extract, and 1% malt extract for 3 days. The *Bam*HIBAC library was constructed by Wuhan Eightstars Bio-Technology Co., Ltd. as described (Luo et al., 2001; Luo and Wing, 2003; Shi et al., 2011). The culture was centrifuged at 4,000 rpm for 10 min to remove the supernatant, and the mycelium was collected for the genomic DNA plug preparation. The restriction enzyme *Bam*HI was used to digest the genome partially, and the fragments with appropriate size were selected. The pMSBBAC1 vector and DH10B *E. coli* host were used to construct a bacterial artificial chromosome (BAC) library with 20-fold genome coverage and around 110 ± 5 kb average inserts. The BAC library totally contained 2,304 clones in six plates (384 clones in each plate). The BAC clones containing the albofungin biosynthetic gene cluster were screened using library-screening primers (Supplementary Table S1). The primers were chosen in both ends and the middle of the gene cluster according to antiSMASH prediction, and the genomic DNA of *S. chrestomyceticus* BCC 24770 was used as the template for PCR positive control. The positive clones were picked, and their plasmids were further extracted for transformation and conjugation.

### Heterologous Expression and LC/MS Detection

The BAC (120 kb) plasmid 4 L19 was introduced into *E. coli* ET12567/pUZ8002 by electroporation and conjugated to *S. coelicolor*. *S. coelicolor* was grown on MS agar (2% mannitol, 2% soya flour, 2% agar, and 10 mM MgSO_4_) to generate spores. The conjugation process was described as follows. *Eschericia coli* ET12567/pUZ8002 containing BAC plasmid 4 L19 was cultured in LB medium with 50 μg ml^−1^ kanamycin, 50 μg ml^−1^ apramycin, and 25 μg ml^−1^ chloramphenicol. Then, 1% of the overnight culture was inoculated into 5 ml LB medium (50 μg ml^−1^ kanamycin, 50 μg ml^−1^ apramycin, and 25 μg ml^−1^ chloramphenicol) to grow until an OD_600_ of 0.4–0.6 was reached. Cultures were centrifuged at 4,000 rpm for 5 mins, and the supernatant was discarded. Cell pellets were washed twice with LB medium and resuspended in 500 μl LB medium. Spores were added into the LB medium at a final volume of 500 μl and heated at 50°C for 10 min. *Escherichia coli* ET12567/pUZ8002 cells with BAC plasmid 4 L19 and the spores were mixed and poured onto the ISP Medium No. 4 agar plate. Plates were incubated at 30°C for 16–18 h, and the surface of the plates was overlaid with 1 ml sterile water (containing 0.5 mg nalidixic acid and 1.25 mg apramycin). Plates were incubated for another 2 days until the conjugants could be picked for growth on the selection plates. The genomic DNA of the conjugants was extracted and screened using library-screening primers (Supplementary Table S1). Positive conjugants were picked and fermented in the AM5 medium for 5 days. The cultured mycelium and broth were extracted with ethyl acetate, and the extracts were evaporated to perform the UPLC/MS analysis.

## Results

### Structure Elucidation

**Figure 1:**
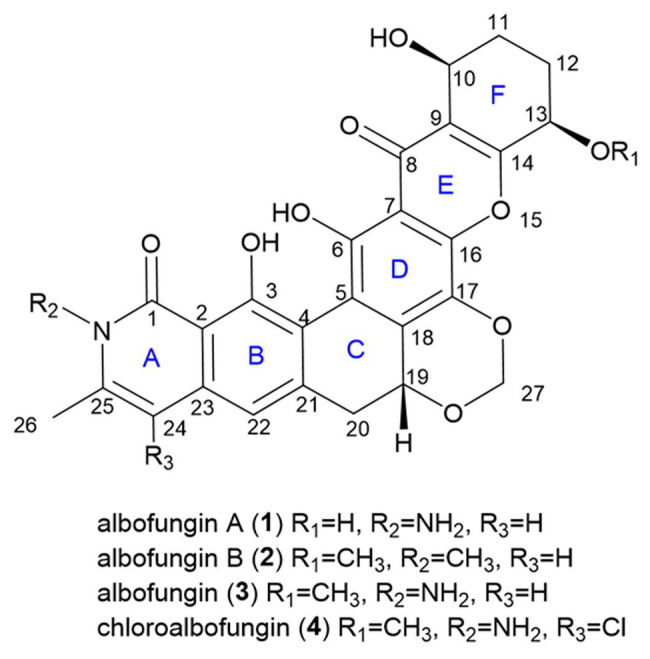
Structures of compounds 1–4.

There was a total of four compounds (1–4) isolated from *S. chrestomyceticus* BCC 24770 from the crude extract. Compound 1 was isolated as a yellow amorphous powder, and its molecular formula was C_26_H_22_N_2_O_9_ on the basis of the high-resolution mass spectroscopy data [*m/z* 507.1389, calculated for C_26_H_23_N_2_O_9_, (M + H)^+^]. By comparing the differences of the ^1^H and ^13^C NMR spectra of 1 and 3 (Table 1), most resonances were quite similar except the resonances of a methoxy group (*δ*_H_ 3.56 and *δ*_C_ 57.8) connected with C-13 in 3 was missing in 1, which indicated that the substitution of C-13 was a hydroxy group instead of a methoxy group. Further, extensive 2D NMR spectroscopic analysis (Figure 1) confirmed the planar structure of 1.

**Figure 2:**
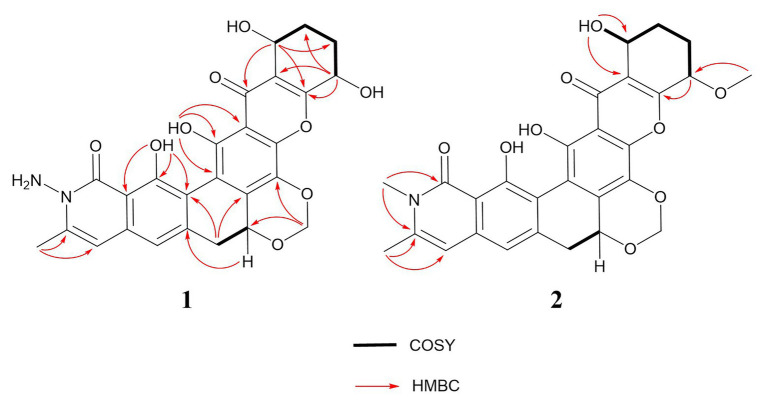
^1^H-^1^H COSY and key HMBC correlations of compounds 1 and 2.

Compound 2 was isolated as a yellow powder and found close to 3 in the LC-MS profile considering its odd molecular weight. This finding suggested the odd number of nitrogen atoms in the formula. The molecular formula of 2 was found to be C_28_H_25_NO_9_ on the basis of the HRESIMS ion at *m/z* 520.1598 (M + H)^+^ (calculated for C_28_H_26_NO_9_). Considering that the UV pattern of 2 was identical to that of 3, we supposed that they share a similar chromophore and backbone. An additional single methyl (*δ*_H_ 3.54 and *δ*_C_ 30.3) in 2 was observed by comparing the ^1^H and ^13^C NMR spectra of 2 and 3 (Table 1). Considering the chemical shift of the additional methyl, we assumed that the methyl was connected to a nitrogen atom. This assumption was confirmed by the key HMBC correlations from the methyl proton to C_1_ (*δ*_C_ 166.1) and C_25_ (*δ*_C_ 141.5). In addition, the analysis of the other HMBC correlations finally established the planar structure of 2 (Figure 2).

**Figure 3:**
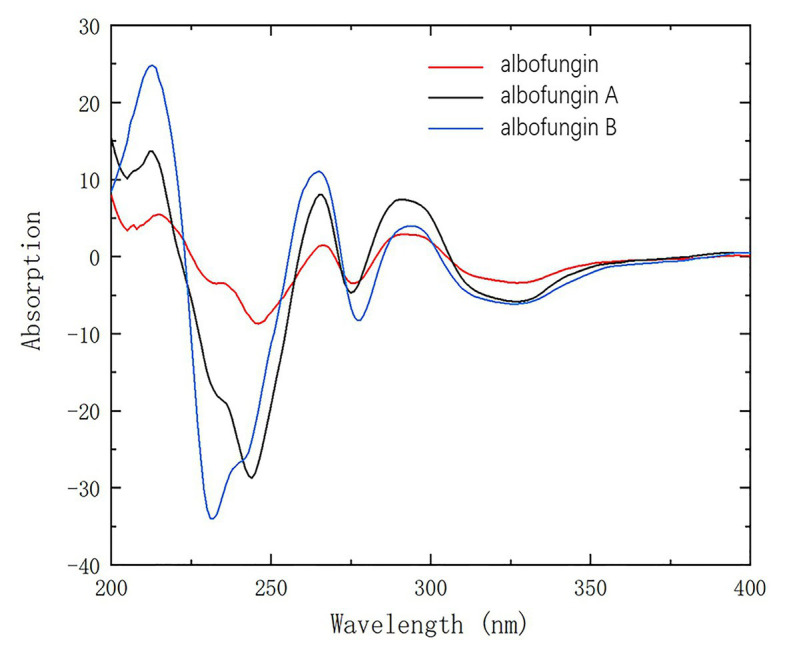
CD spectra of compounds 1 and 2 compared with 3 in methanol.

Given that they shared a similar biosynthesis pathway with 3 and 4, 1 and 2 might reasonably possess the same stereochemistry as 3 and 4. The absolute configurations of 3 and 4 were established as 10-*S*, 13-*R*, and 19-*R* by using the X-ray crystallographic analysis in our previous study (Ye et al., 2020). The circular dichroism experiment was conducted to verify the stereochemistry of 1 and 2. The experimental electronic circular dichroism spectra showed that 1 and 2 exhibited a similar pattern with 3. The negative cotton effects were at 220–250, 271–280, and 305–380 nm regions, and positive cotton effects were at 200–220, 258–271, and 284–303 nm regions. Therefore, the absolute stereochemistries of 1 and 2 were determined as 10-*S*, 13-*R*, and 19-*R* (Figure 3).

### Antibacterial and Antitumor Activities of Albofungin Derivatives

We examined the isolated albofungin derivatives for their bioactivities against most of the notorious ESKAPE bacteria, including *S. aureus*, *K. pneumoniae*, *A. baumannii*, and *E. cloacae*, which are the leading causes of healthcare-associated infections around the world (Santajit and Indrawattana, 2016). All compounds (1–4) exhibited strong bioactivities at a nanomolar range on the tested Gram-positive bacteria (Table 2). The compounds with a rare N-aminoamide linked in the A ring having better activities (1, 3, and 4 vs. 2) suggested that the existence of N-aminoamide was essential for their bioactivities against Gram-positive bacteria.

**Table 2:** Minimal inhibition concentration (MIC) of albofungin compounds (1–4) against pathogenic Gram-positive bacteria.

	Anti-Gram-positive bacterial activities (MIC, nM)			
	Compound 1	Compound 2	Compound 3	Compound 4
MRSA ATCC 43300	<0.1	30	<0.1	0.1
*Staphylococcus aureus* ATCC 25923	0.4	30	<0.1	0.1
*Staphylococcus aureus* B04	6.3	30	<0.1	3.6
*Bacillus subtilis* ZK31	<0.1	<0.1	<0.1	0.1

Vancomycin was used as a positive control.

Among the tested compounds, 1 showed the most potent activities toward Gram-negative bacteria, including *K. pneumoniae* NRRL-B-3521, *K. pneumoniae* NRRL-B-408, *A. baumannii* B-65371, *E. cloacae* NRRL-B-425, *E. coli* k12, and *E. coli* MG1655 at a low micromolar range (Table 3). We suspected that 1 could penetrate the outer membrane of bacteria easier due to the hydroxyl group in the F ring rather than a methoxy group compared with the 3.

**Table 3:** MIC of albofungin compounds (1–4) against pathogenic Gram-negative bacteria.

Anti-Gram-negative bacterial activities (MIC, μM)				
	1	2	3	4
*Klebsiella pneumoniae* NRRL-B-3521	0.16	>38	0.77	>36
*Klebsiella pneumoniae* NRRL-B-408	0.16	>38	0.77	>36
*Acinetobacter baumannii* B-65371	0.32	7.7	7.7	>36
*Enterobacter cloacae* NRRL-B-425	0.32	>38	7.7	>36
*Escherichia coli* k12	0.32	>38	1.5	36
*E. coli* MG1655	0.79	>38	1.5	>36

Kanamycin was used as a positive control.

Moreover, we tested the antitumor activities of albofungin derivatives toward HeLa (cervical carcinoma), MCF 7 (breast carcinoma), and HepG2 (hepatocellular carcinoma) cells. All compounds displayed significant antitumor activities with the IC_50_ ranging from 0.003 μM to 0.9 μM (Table 4).

**Table 4:** IC_50_ of albofungin compounds (1–4) against human cancer cell lines.

IC_50_ (μM)			
	HeLa (cervix)	MCF 7 (breast)	HepG2 (liver)
1	0.003	0.005	0.02
2	0.016	0.012	0.33
3	0.008	0.006	0.038
4	0.018	0.007	0.9

Cisplatin was used as a positive control.

### Induction of Apoptosis in HeLa and MCF 7 Cells by Compound 1

**Figure 4:**
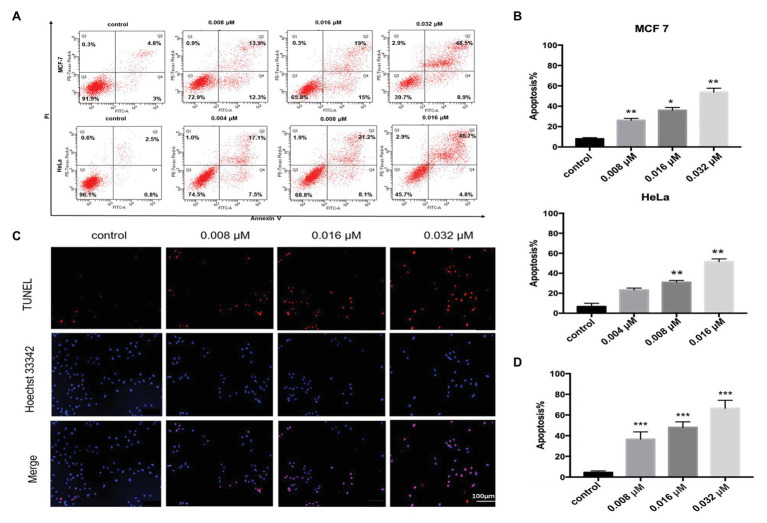
Compound 1-induced apoptosis in HeLa and MCF 7 cells. **(A)** Flow cytometry analysis of HeLa and MCF 7 cells treated with or without compound 1 for 24 h and stained with FITC–Annexin V/PI. Distribution of necrotic (i.e., Q1, annexin V−, and PI+), late apoptotic (i.e., Q2, annexin V+, and PI+), viable (i.e., Q3, annexin V−, and PI−), and early apoptotic (i.e., Q4, annexin V+, and PI−) cells. **(B)** Quantitative result of apoptosis rate in HeLa and MCF 7 cells analyzed using flow cytometry. Data were presented as mean ± SD from three independent experiments. ^*^p < 0.05, ^**^p < 0.01, and ^***^p < 0.001 compared with the control group. **(C)** HeLa cells treated with compound 1 for 24 h and stained with TUNEL (red) to detect apoptotic cells. The nucleus was stained with Hoechst 33342 (blue). **(D)** Quantitative result of apoptosis rate in HeLa cells analyzed using the TdT-mediated deoxynucleotidyl transferase (dUTP) nick end labeling (TUNEL) assay. Data were presented as mean ± SD from three independent experiments. ^*^p < 0.05, ^**^p < 0.01, and ^***^p < 0.001 compared with the control group.

Polycyclic xanthone compounds had very potent antitumor activities toward various cancer cell lines. Among the isolated albofungin derivatives, 1 exhibited the lowest IC_50_ value toward HeLa cells in a dose- and time-dependent manner. Thus, the cell death mechanism behind this compound was further investigated. The Annexin V/PI double staining and flow cytometry analysis were performed to determine whether 1 could trigger apoptosis in HeLa and MCF 7 cells. After 24 h treatment of 1, the apoptosis rate significantly increased from 7.8 to 57.4% in MCF 7 cells and from 3.3 to 48.6% in HeLa cells in a dose-dependent manner (Figures 4A,B).

Furthermore, we used the TUNEL assay to verify the results. The TUNEL assay can detect the DNA fragmentation generated from double-strand DNA breaks in cells undergoing apoptosis by labeling the 3′-hydroxyl termini. The Hoechst 33342 was used to detect all apoptotic or nonapoptotic cells. After 24 h of treatment, the apoptosis cell rate was approximately 36.6% at 0.008 μM concentration and nearly 66.5% at 0.032 μM concentration (Figures 4C,D). This data indicated that 1 could trigger apoptosis in HeLa and MCF 7 cells in a dose-dependent manner.

### Proposed Albofungin Biosynthesis Pathway

**Figure 5:**
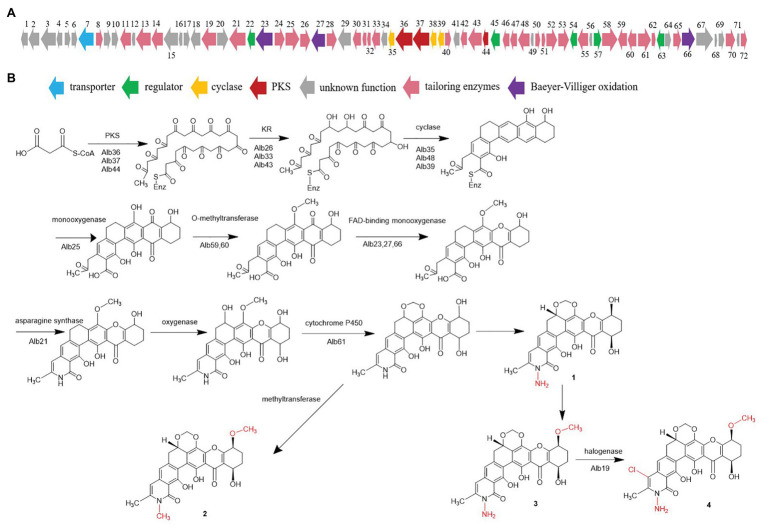
**(A)** Gene cluster of albofungin from BCC 24770 and **(B)** proposed albofungin biosynthesis pathway.

Considering the chemical structures of the isolated albofungin derivatives, we proposed their biosynthesis pathway (Figure 5). The draft genome of *S. chrestomyceticus* was sequenced to support our hypothesis. A total of 17,075,412 clean reads were used to assemble a draft genome with 70 contigs of 9.34 Mb (100% genome completeness). The draft genome predicted 7,906 genes. The antiSMASH analysis for the whole genome predicted 41 biosynthetic gene clusters (Supplementary Table S3), including two Type II polyketide synthases (PKSs) biosynthetic gene clusters. One of the Type II PKS biosynthetic gene clusters (8.1) showed 48% similarity to the xantholipin (MIBiG accession: BGC0000279) and the lysolipin I (MIBiG accession: BGC0000242) biosynthetic gene clusters, which consisted of 72 open reading frames (Supplementary Table S4), including a minimal PKS gene, modification tailoring genes, regulator, and transporter genes.

The acyl-CoA carboxylase (Alb51, Alb58) might catalyze carboxylation of acetyl-CoA to malonyl-CoA, which begins the biosynthesis pathway. The biosynthesis study of polycyclic xanthone FD549 and lysolipin revealed that the polyketide chain was formed by the catalysis of type II PKS (Lopez et al., 2010; Kudo et al., 2011). Thus, the existence of a minimal PKS gene set coding for a ketosynthase α (Alb37), a ketosynthase β/chain length factor (Alb36), and an acyl carrier protein (Alb44) is supposed to be responsible for condensation of 13 malonate and synthesizing a polyketide precursor. Cyclases (Alb35, Alb38, and Alb39) then catalyzed the cyclization of the extended linear polyketide chain. The Baeyer–Villiger oxidation was involved in the xanthone scaffold formation reported by the biosynthesis of lysolipin I and FD 549. Among the three FAD-dependent monooxygenases (i.e., Alb23, Alb27, and Alb66), Alb23 showed 80.41, 70.73, and 42.2% to XanO4, LlpOVIII, and PnxO4, respectively. The P450 monooxygenase (Alb61) was responsible for generating the methylenedioxy bridge, and an asparagine synthetase homolog (Alb21) was involved in the amide ring formation. A chlorine atom substituted on ring A was catalyzed by the halogenase. Bromine was introduced to confirm the function of halogenase, and we detected the characteristic bromoalbofungin signal right close to the 4 on the LCMS profile (Supplementary Figure S21) in the fermentation of *S. chrestomyceticus* BCC 24770 (0.01% potassium bromide, 0.4% glucose, 0.4% yeast extract, and 1% malt extract). Four regulatory genes (i.e., *alb*22, *alb*45, *alb*57, and *alb*63) and one transporter gene (*alb*7) were assumed to participate in the biosynthesis of albofungins.

### Heterologous Expression of the Albofungin Biosynthetic Gene Cluster

**Figure 6:**
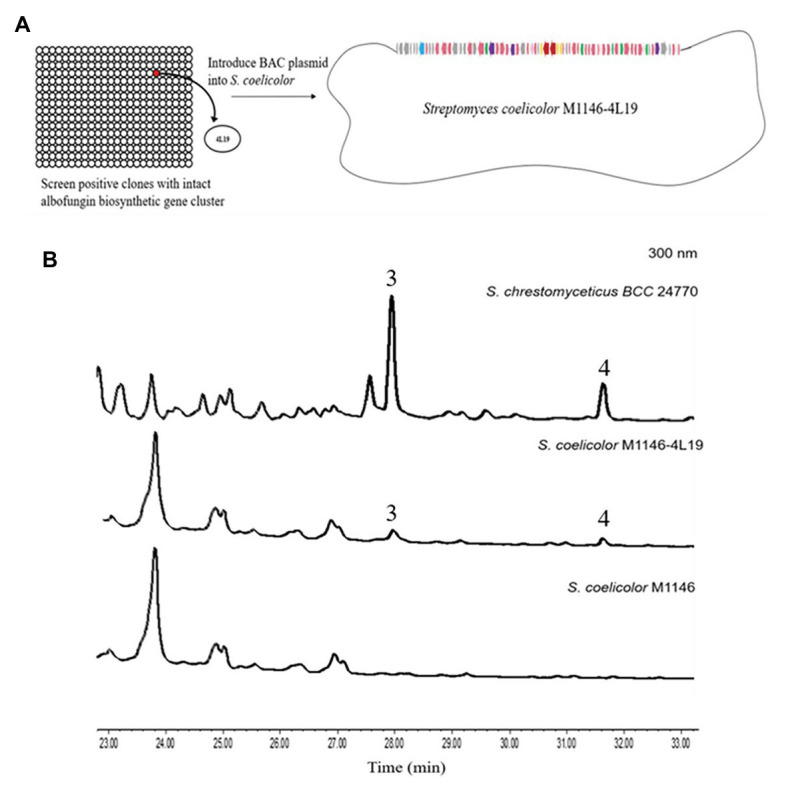
**(A)** Construction of the bacterial artificial chromosome (BAC) library and screening for clones containing the entire albofungin biosynthetic gene cluster and heterologous expression of BAC-4 L19 in *Streptomyces coelicolor*. **(B)** HPLC profiles of the metabolites of *Streptomyces chrestomyceticus* BCC24770 and recombinant strains.

We used the genomic DNA of *S. chrestomyceticus* BCC 24770 for BAC library construction (total 2,304 clones) to identify the biosynthetic gene cluster for albofungin derivatives. We used three pairs of primers (i.e., Lib-screen-up F/R, Lib-screen-middle F/R, and Lib-screen-down F/R) to perform PCR screening for the clones, including the middle and left/right boundary region, and obtain the entire gene cluster. One positive clone 4 L19 was selected (Supplementary Figure S22). The plasmid 4 L19 was further transformed into the *S. coelicolor* M1146 for heterologous expression. We randomly selected eight conjugants from each heterologous host grown on the ISP Medium No. 4 plate with apramycin and nalidixic acid to perform a three-round PCR screening. The conjugants successfully integrated with intact albofungin biosynthetic gene clusters were fermented, and their secondary metabolites were extracted. We compared the HPLC and the UPLC-MS results of the metabolite profiles of mutant strains and wild-type heterologous hosts and found that two new peaks appeared in the mutant strain *S. coelicolor* M1146-4 L19 metabolites (Figure 6; Supplementary Figure S23). The retention times of these two new peaks were the same with those of the 3 and 4. We further confirmed the compounds using their UV patterns and exact masses. Therefore, the albofungin biosynthetic gene cluster was verified in the genome of *S. chrestomyceticus*.

## Discussion

The compounds from the polycyclic xanthone family, such as simaomicin, cervinomycin, and xantholipin, were reported to have strong activities against Gram-positive bacteria and antitumor activities on various cancer cell lines (Terui et al., 2003; Koizumi et al., 2009; Hu et al., 2020). Sharing an oxygenated angular hexacyclic backbone containing a rare N-aminoamide, 3 and its derivatives show potent antibacterial activity against a broad spectrum of Gram-positive and Gram-negative pathogenic bacteria, including *K. pneumoniae*, *A. baumannii*, *E. cloacae*, and *S. aureus*. The albofungin is a bacterial transglycosylase inhibitor (Wu et al., 2018). Therefore, the discovery of novel albofungin derivatives and their structure-activity relationship analysis provided instructions for chemical modifications of the albofungin backbone to generate derivatives with improved bioactivities.

The heterologous expression has achieved significant advances in the genomic era, promoting the discovery of novel natural products and paving the way for validating the functions of the entire biosynthesis pathway in an engineered host (Luo et al., 2016; Tu et al., 2016). This strategy enables the improved study of the gene functions in the pathway through genetic mutations (Huo et al., 2019). The BGCs of polyketides are usually larger than 40 kb. Therefore, using the BAC vectors to construct genomic libraries is an efficient approach for capturing these large BGCs (Xu et al., 2016). In this study, the BAC library construction remarkably achieved large BGCs capturing and heterologous expression in the *S. coelicolor* M1146, which provides opportunities to understand the unique post-PKS modifications in the albofungin biosynthesis.

## Conclusion

In conclusion, our work reported the isolation, purification, and structure elucidation of two novel albofungin derivatives, 1 and 2 from *S. chrestomyceticus*. Their antibacterial activity was evaluated, and the antitumor mechanism was further explored. Briefly, all compounds exhibited potent antibacterial and antitumor activities. Compound 1 showed activities against Gram-positive and Gram-negative pathogenic bacteria from the micromolar range to the nanomolar range. Also, the working mechanism of the antitumor activity of these compounds was proven through the upregulated apoptosis in HeLa and MCF 7 cells. These results suggested that albofungin derivatives could be potential candidates for the development of broad-spectrum antibiotics and antitumor drugs. We also conducted the genome-based analysis and heterologous expression of the gene cluster encoding the biosynthesis of albofungins. Based on the characterization of the albofungin biosynthesis pathway, we hypothesized that the minimal PKS genes (i.e., *alb*36, *alb*37, and *alb*44), regulator, and transporter genes (i.e., *alb*7, *alb*22, *alb*45, *alb*57, and *alb*63) and other tailoring genes participated in the albofungin biosynthesis. Therefore, further genetic characterization work is required to test this hypothesis and decipher unusual modifications, such as the involvement of N-aminoamide in the biosynthesis of albofungins.

## Data Availability Statement

The datasets presented in this study can be found in online repositories. The names of the repository/repositories and accession number(s) can be found below: CCDC, Cambridge Crystallographic Data Centre – 2044031, 2044030; NCBI – PRJNA682011.

## Author Contributions

WS and P-YQ designed the experiments. WS and WY performed experiments. AC, XL, and YL analyzed the data. AC, JT, WY, and FC revised the manuscript. WS wrote the manuscript. All authors contributed to the article and approved the submitted version.

### Conflict of Interest

The authors declare that the research was conducted in the absence of any commercial or financial relationships that could be construed as a potential conflict of interest.

## Footnotes

^1^http://antismash.secondarymetabolites.org/
