# Supplementary material for: Discovery, Bioactivity Evaluation, Biosynthetic Gene Cluster Identification, and Heterologous Expression of Novel Albofungin Derivatives
Source: Front Microbiol. 2021 Feb 1;12:635268. doi: 10.3389/fmicb.2021.635268 (PMC7902042; doi:10.3389/fmicb.2021.635268)
Supplement: Supplementary file 1 [file Data_Sheet_1.pdf]

Supplementary Material

Discovery, bioactivity evaluation, biosynthetic gene cluster identification and heterologous expression of novel albofungin derivatives

Weiyi She[1,2,3], Wenkang Ye[1,2,3], Aifang Cheng[2,3], Xin Liu[2,3], Jianwei Tang[2,3], Yi Lan[2,3], Feng Chen[4] and Pei-Yuan Qian[2,3*]

[1]SZU-HKUST Joint Ph.D. Program in Marine Environmental Science, Shenzhen University, Shenzhen, China
[2]Hong Kong Branch of the Southern Marine Science and Engineering Guangdong, Laboratory (Guangzhou), Hong Kong University of Science and Technology, Clear Water Bay, Hong Kong, People's Republic of China
[3]Division of Ocean Science, Hong Kong University of Science and Technology, Clear Water Bay, Hong Kong, People's Republic of China
[4]Institute for Advanced Study, Shenzhen University, 518060, Shenzhen, China

*** Correspondence:**
Pei-Yuan Qian
boqianpy@ust.hk

Supplementary Table 1. Primers used in this study.

Primers' name	Sequences (5'-3')
**BAC plasmid screening (4L19)**	
Library-screening-up-F	gcgagcatgctgttgtactt
Library-screening-up-R	ggtggcagaaatggatagcc
Library-screening-middle-F	tcgtcgtccagcagtgcgta
Library-screening-middle-R	gtactgttcacgtcctgggc
Library-screening-down-F	caaggtgcactgccctctgg
Library-screening-down-R	gcgatctccacgggcgagta

PCR reaction is as follow: 95°C for 5 min; 30 cycles each of 95°C for 30 s, 65°C for 30 s and 72°C for 70 s; 72°C for 5 min by using 2×Taq PCR Mastermix (KT201) with 6% DMSO.

Supplementary Table 2. Strains and plasmids used in this study.

Strain	Genotype description	Source
E. Coli strains		
E. Coli Top 10	*E. Coli* host for cloning	Commercial
E. Coli ET12567/pUZ8002	*E. Coli* host for conjugation	Commercial
Streptomyces strains		
Streptomyces coelicolor	Heterologous expression strain	Commercial
Streptomyces chrestomyceticus BCC 24770	Albofungin derivatives producing strain	Commercial
Plasmids		
pHZAUBACFXJ1	BAC library vector, Apr^R	Commercial

Supplementary Table 3. AntiSMASH analysis for biosynthetic gene clusters in the draft genome sequences of *Streptomyces chrestomyceticu*s BCC 24770.

NO.	Type	Most similar known cluster	Similarity	MIBiG accession
1.1	ectoine	ectoine	100%	BGC0000853
1.2	terpene	geosmin	100%	BGC0001181
1.3	lanthipeptide	SapB	75%	BGC0000551
1.4	CDPS	purincyclamide	100%	BGC0001986
2.1	phosphonate	phosphinothricintripeptide	17%	BGC0000406
2.2	transAT-PKS, NRPS-like	9-methylstreptimidone	19%	BGC0000171
2.3	Arylpolyene, terpene	herboxidiene	4%	BGC0001065

2.4	terpene	hopene	76%	BGC0000663
2.5	NRPS	isocomplestatin	87%	BGC0000326
2.6	terpene	kanamycin	4%	BGC0000703
2.7	other	formicamycins A-M	20%	BGC0001590
2.8	bacteriocin			
2.9	butyrolactone			
3.1	NRPS, PKS-like	tyrobetaine	86%	BGC0001813
4.1	linaridin	legonaridin	66%	BGC0001188
4.2	T2PKS	lugdunomycin	22%	BGC0002016
5.1	NRPS, T1PKS, lassopeptide	thiazostatin / watasemycin A / watasemycin B / 2-hydroxyphenylthiazoline enantiopyochelin / isopyochelin	86%	BGC0001801
5.2	terpene			
6.1	NRPS, NRPS-like, T1PKS	mannopeptimycin	14%	BGC0000388
6.2	terpene			
6.3	NRPS	paenibactin	33%	BGC0000401
6.4	phosphonate	dehydrophos	11%	BGC0000897
6.5	NRPS-like, betalactone	ketomemicin B3 / ketomemicin B4	33%	BGC0001633
6.6	NRPS, T1PKS	pentamycin	33%	BGC0002032
7.1	NRPS, T1PKS	malonomycin	88%	BGC0001942
7.2	terpene	ebelactone	5%	BGC0001580
7.3	NRPS	deimino-antipain	66%	BGC0001570
8.1	Lassopeptide, T2PKS, NRPS	xantholipin	48%	BGC0000279
8.2	NRPS, thiopeptide, LAP, terpene	cyclothiazomycin C	100%	BGC0001146
8.3	NRPS-like, lassopeptide, NRPS	lagmysin	80%	BGC0001645
8.4	Amglyccycl, NRPS-like, other	paromomycin	82%	BGC0000712
9.1	butyrolactone	neocarzinostatin	6%	BGC0000112
10.1	bacteriocin			
10.2	NRPS,	isorenieratene	75%	BGC0001456

	T1PKS, terpene			
12.1	siderophore			
12.2	NRPS	mannopeptimycin	81%	BGC0000388
12.3	NRPS, lanthipeptide	streptobactin	70%	BGC0000368
14.1	siderophore	desferrioxamine E	100%	BGC0001478
14.2	siderophore			
15.1	lassopeptide	SSV-2083	18%	BGC0000579
18.1	NRPS-like, T1PKS	lasalocid	14%	BGC0000087

Supplementary Table 4. Predicted functions of open reading frames in the albofungin biosynthetic gene cluster of *Streptomyces chrestomyceticu*s BCC 24770.

Gene	Size (AA)	Proposed function	Protein homolog	Identity/Positive (%)	Protein accession
1	907	fibronectin type III domain-containing protein	*Streptomyces mobaraensis*	73/74	WP_152262558.1
2	868	fibronectin type III domain-containing protein	*Streptomyces* sp. TYQ1024	90/94	WP_185943483.1
3	907	fibronectin type III domain-containing protein	*Streptomyces mobaraensis*	84/90	WP_152262558.1
4	222	hypothetical protein	*Streptomyces* sp. GY16	36/48	WP_152169605.1
5	97	STAS domain-containing protein	*Streptomyces rimosus*	47/49	WP_033027222.1
6	309	sensor histidine kinase	*Streptomyces rimosus*	82/87	WP_030374215.1
7	501	MFS transporter	*Streptomyces monomycini*	96/97	WP_106968724.1
8	107	antibiotic biosynthesis monooxygenase	*Streptomyces monomycini*	100/100	WP_030019001.1
9	224	hypothetical protein	*Streptomyces monomycini*	89/92	WP_033037348.1
10	224	hypothetical protein	*Streptomyces monomycini*	95/95	WP_033037348.1
11	237	short-chain dehydrogenase	*Streptomyces* sp. NRRL WC-3618	72/79	KOV60939.1
12	126	hypothetical protein	*Streptomyces monomycini*	94/96	WP_050502249.1

13	460	FAD-binding oxidoreductase	*Streptomyces monomycini*	95/97	WP_050502251.1
14	369	FAD-binding oxidoreductase	*Streptomyces monomycini*	92/94	WP_050502252.1
15	457	class I tRNA ligase family protein	*Streptomyces monomycini*	93/95	WP_063726047.1
16	116	cupin domain-containing protein	*Streptomyces monomycini*	92/98	WP_030019008.1
17	159	hypothetical protein	*Streptomyces monomycini*	94/94	WP_157851505.1
18	518	right-handed parallel beta-helix repeat-containing protein	*Streptomyces monomycini*	87/92	WP_030019010.1
19	583	tryptophan 7-halogenase	*Streptomyces monomycini*	93/96	WP_050502253.1
20	452	type III glutamate–ammonia ligase	*Streptomyces monomycini*	96/97	WP_030019012.1
21	621	asparagine synthase (glutamine-hydrolyzing)	*Streptomyces monomycini*	98/99	WP_030019013.1
22	255	TenA family transcriptional regulator	*Streptomyces morookaense*	69/74	WP_171079302.1
23	536	FAD-dependent monooxygenase	*Streptomyces* sp. NRRL WC-3618	80/88	WP_053740214.1
24	337	methyltransferase	*Streptomyces monomycini*	97/99	WP_030019016.1
25	442	SidA/IucD/PvdA family monooxygenase	*Streptomyces monomycini*	96/97	WP_050502255.1
26	250	3-oxoacyl-(acyl-carrier protein) reductase	uncultured bacterium	49/62	AEM44279.1
27	399	FAD-dependent monooxygenase	*Streptomyces monomycini*	93/95	WP_050502266.1
28	284	LLM class flavin-dependent oxidoreductase	*Streptomyces monomycini*	98/98	WP_030019020.1
29	401	DUF1205 domain-containing protein	*Streptomyces monomycini*	95/96	WP_030019021.1
30	287	NAD(P)-dependent oxidoreductase	*Streptomyces monomycini*	95/97	WP_030019022.1
31	103	antibiotic biosynthesis monooxygenase	*Streptomyces monomycini*	95/98	WP_030019023.1
32	107	antibiotic biosynthesis monooxygenase	*Streptomyces monomycini*	94/98	WP_030019024.1
33	250	putative 3-oxoacyl-ACP reductase	*Streptomyces flavogriseus*	75/86	ADE22311.1

34	151	DUF1772 domain-containing protein	*Streptomyces monomycini*	97/98	WP_030019026.1
35	153	polyketide cyclase	*Streptomyces monomycini*	94/96	WP_030019027.1
36	410	ketosynthase chain-length factor	*Streptomyces monomycini*	95/97	WP_030019028.1
37	429	beta-ketoacyl-(acyl-carrier-protein) synthase family protein	*Streptomyces monomycini*	97/99	WP_030019029.1
38	144	putative polyketide cyclase	*Streptomyces flavogriseus*	78/84	ADE22316.1
39	111	TcmI family type II polyketide cyclase	*Streptomyces monomycini*	95/99	WP_030019031.1
40	132	SchA/CurD	*Streptomyces monomycini*	95/96	WP_033037350.1
41	150	DUF1772 domain-containing protein	*Streptomyces monomycini*	95/96	WP_030019033.1
42	129	SchA/CurD	*Streptomyces monomycini*	96/97	WP_030019034.1
43	256	3-oxoacyl-(acyl-carrier protein) reductase	*Streptomyces* sp. BK335	73/80	TCS44138.1
44	89	acyl carrier protein	*Streptomyces monomycini*	99/100	WP_030019036.1
45	274	AfsR/SARP family transcriptional regulator	*Streptomyces monomycini*	99/98	WP_030019037.1
46	256	thioesterase	*Streptomyces monomycini*	92/95	WP_050502256.1
47	270	4′-phosphopantetheinyl transferase superfamily protein	*Streptomyces monomycini*	94/96	WP_078624091.1
48	591	acyl-CoA dehydrogenase	*Streptomyces monomycini*	70/76	WP_171079271.1
49	72	hypothetical protein	*Streptomyces monomycini*	93/95	WP_030019041.1
50	224	antibiotic biosynthesis monooxygenase	*Streptomyces monomycini*	83/87	WP_030019042.1
51	71	acyl-CoA carboxylase subunit epsilon	*Streptomyces monomycini*	82/83	WP_030019043.1
52	579	fatty acyl–AMP ligase	*Streptomyces monomycini*	98/98	WP_030019044.1
53	413	acyltransferase domain-containing protein	*Streptomyces monomycini*	92/93	WP_030019045.1
54	204	response regulator transcription factor	*Streptomyces monomycini*	99/99	WP_030019046.1

55	306	histidine kinase	*Streptomyces monomycini*	96/98	WP_167745398.1
56	88	conotoxin	*Conus praecellens*	57/86	ATF27748.1
57	264	AfsR/SARP family transcriptional regulator	*Streptomyces monomycini*	97/97	WP_030019048.1
58	529	acyl-CoA carboxylase subunit beta	*Streptomyces monomycini*	96/97	WP_030019049.1
59	344	*O*-methyltransferase	*Nocardiopsis flavescens*	45/57	SHI64799.1
60	336	*O*-methyltransferase	*Streptomyces chattanoogensis*	59/72	AIM19636.1
61	399	cytochrome P450	*Streptomyces monomycini*	97/98	WP_030019052.1
62	80	ferredoxin	*Streptomyces monomycini*	95/95	WP_050502258.1
63	179	MarR family transcriptional regulator	*Streptomyces monomycini*	93/94	WP_078624093.1
64	198	hypothetical protein	*Streptomyces monomycini*	95/96	WP_030019055.1
65	254	SDR family oxidoreductase	*Streptomyces monomycini*	97/97	WP_030019056.1
66	459	FAD-dependent monooxygenase	*Streptomyces monomycini*	97/98	WP_030019057.1
67	1087	hypothetical protein	*Streptomyces* sp. 604F	63/75	WP_164184022.1
68	125	hypothetical protein	*Streptomyces exfoliatus*	73/85	WP_137994062.1
69	167	hypothetical protein	*Streptomyces* sp. WZ.A104	52/67	WP_096632922.1
70	608	esterase	*Streptomyces chrestomyceticus* JCM 4735	99/99	GCD33035.1
71	74	DUF397 domain-containing protein	*Streptomyces carpinensis*	64/76	WP_086722539.1
72	257	phytanoyl-CoA dioxygenase family protein	*Streptomyces rimosus*	97/98	WP_125051672.1

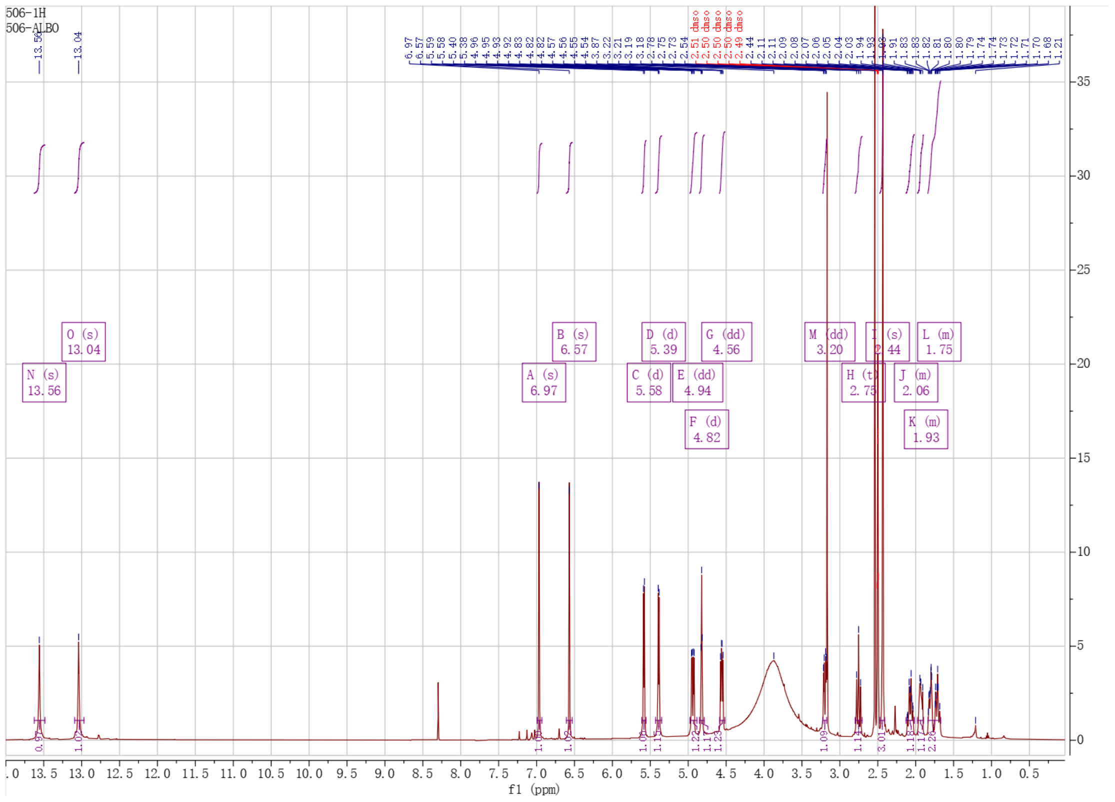

Supplementary Figure 1. ^{1}H NMR spectrum of albofungin A (**1**) in DMSO-d_6.

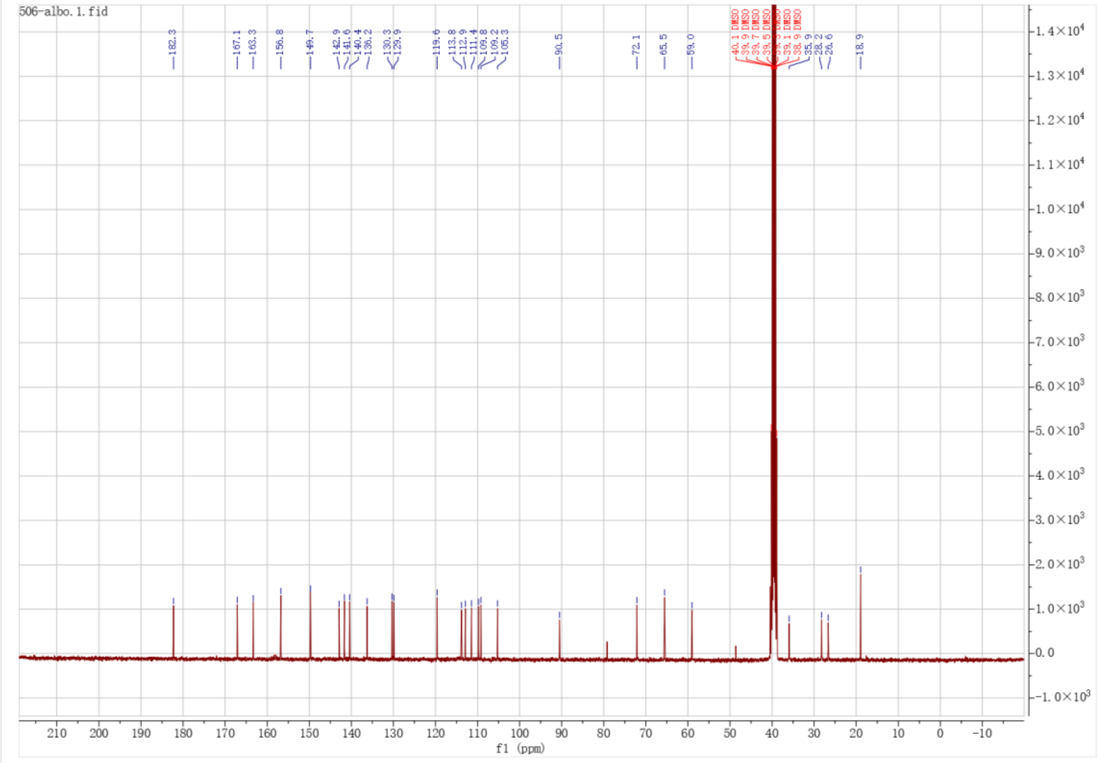

Supplementary Figure 2. ^{13}C NMR spectrum of albofungin A (**1**) in DMSO-d_6.

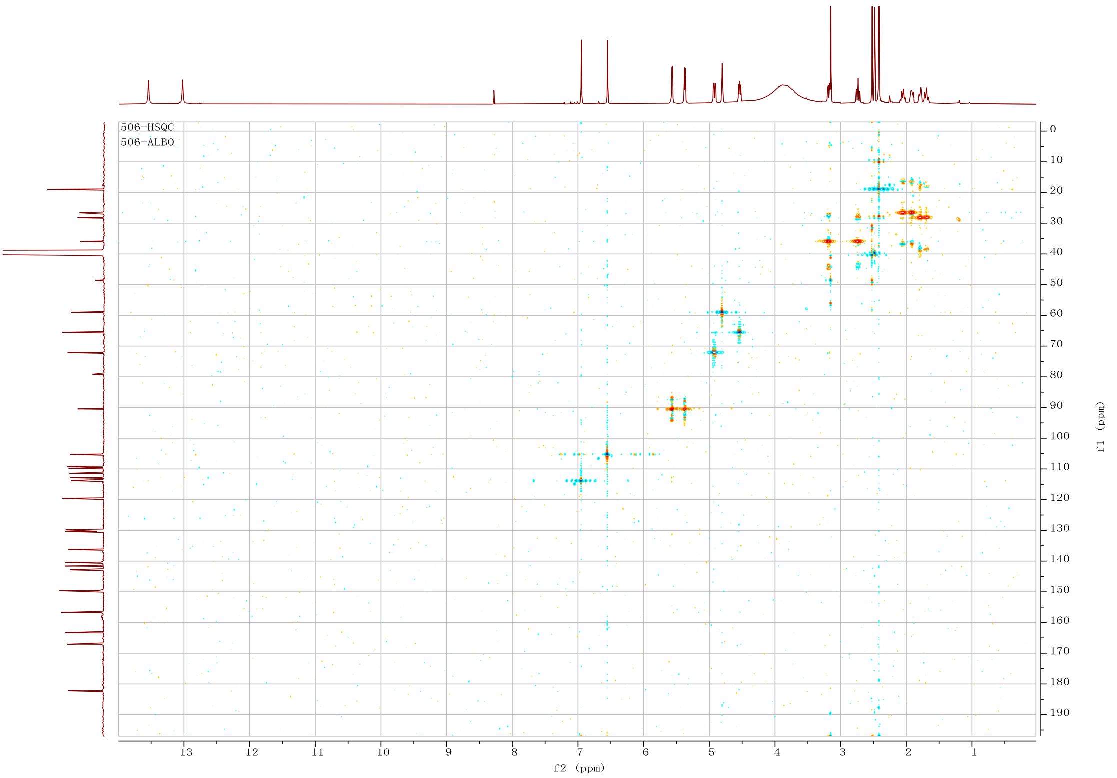

Supplementary Figure 3. HSQC spectrum of albofungin A (**1**) in DMSO-d_6.

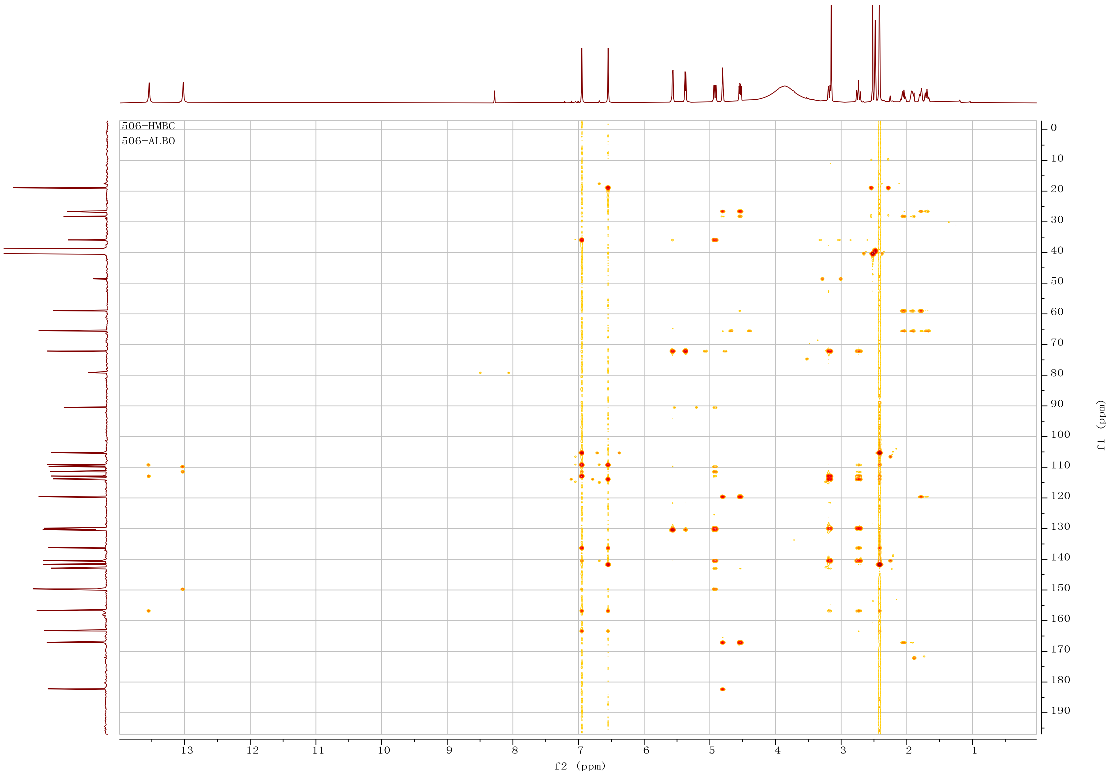

Supplementary Figure 4. HMBC spectrum of albofungin A (**1**) in DMSO-d_6.

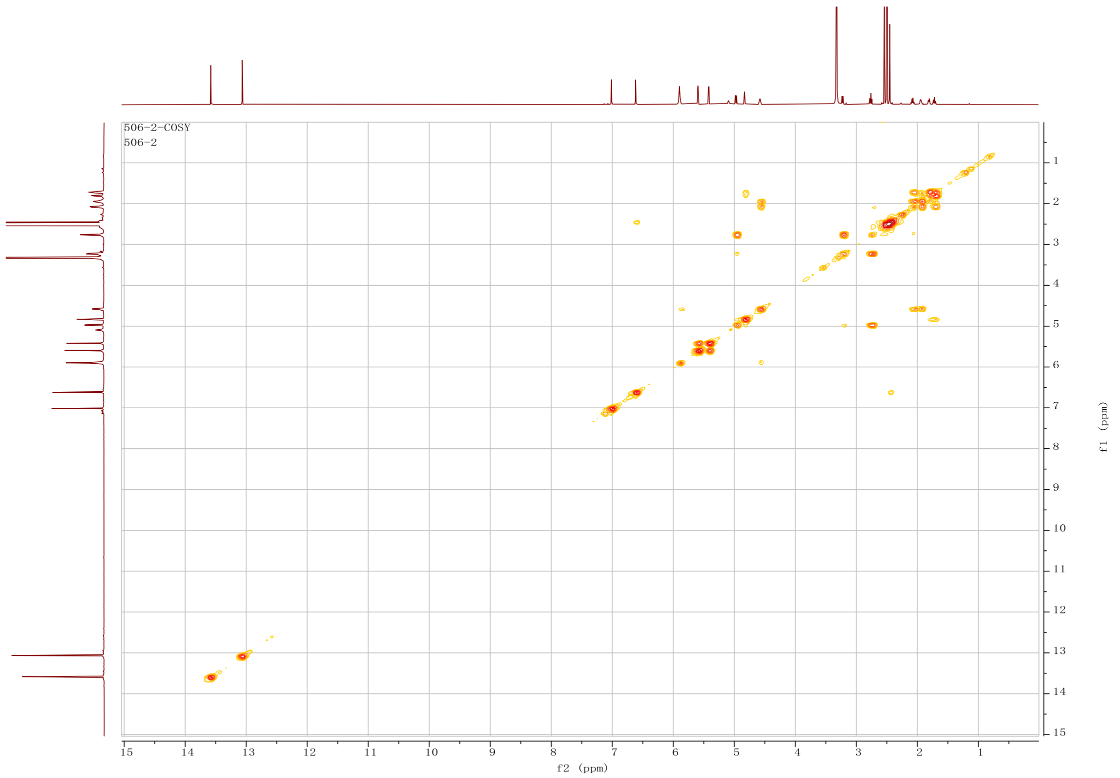

Supplementary Figure 5. COSY spectrum of albofungin A (**1**) in DMSO-d_6.

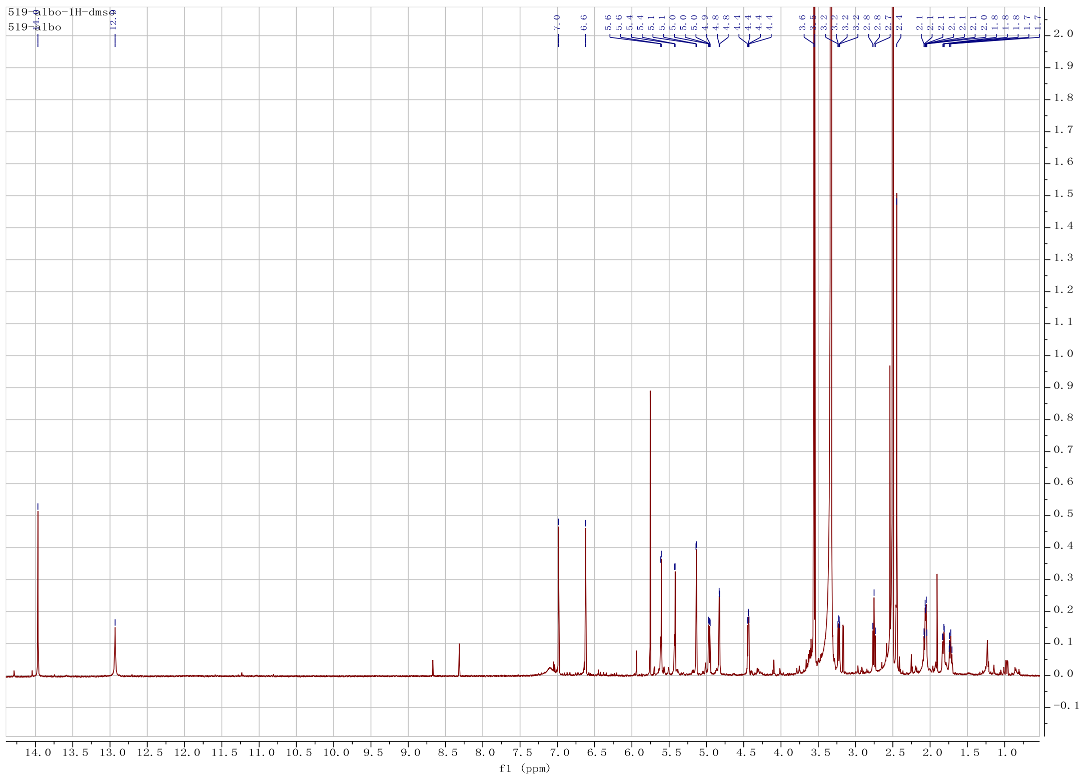

Supplementary Figure 6. ^{1}H NMR spectrum of albofungin B (**2**) in DMSO-d_6.

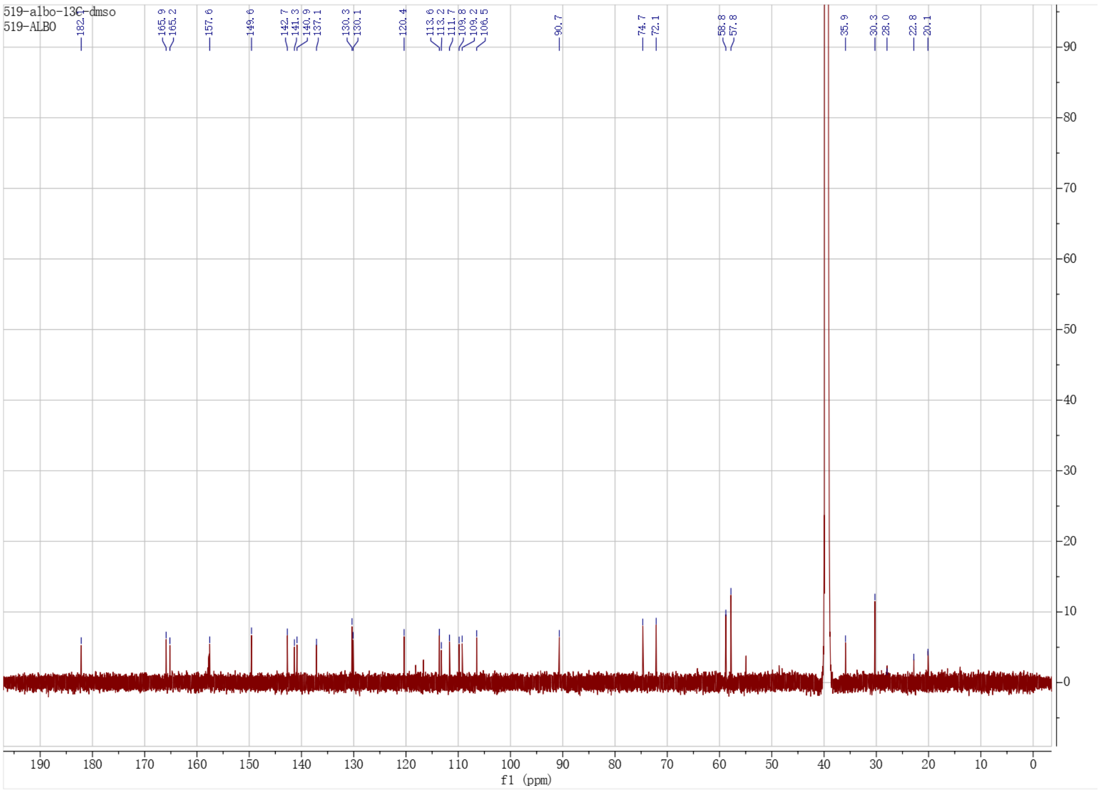

Supplementary Figure 7. ^{13}C NMR spectrum of albofungin B (**2**) in DMSO-d_6.

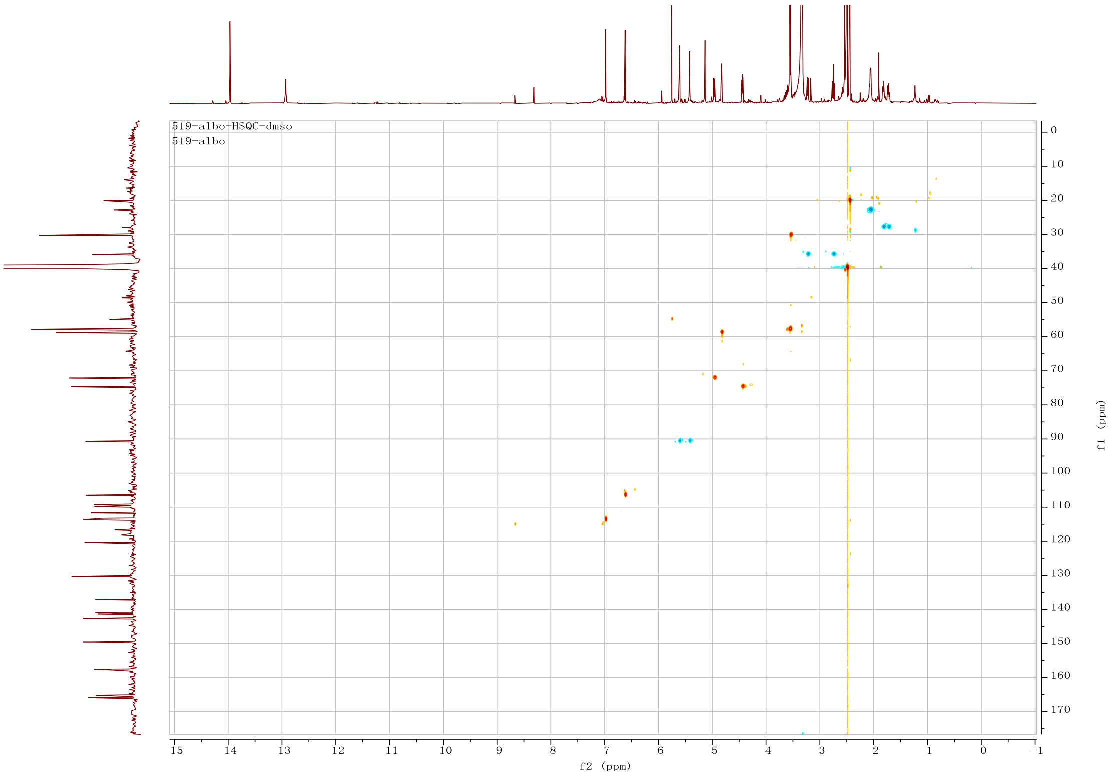

Supplementary Figure 8. HSQC spectrum of albofungin B (**2**) in DMSO-d_6.

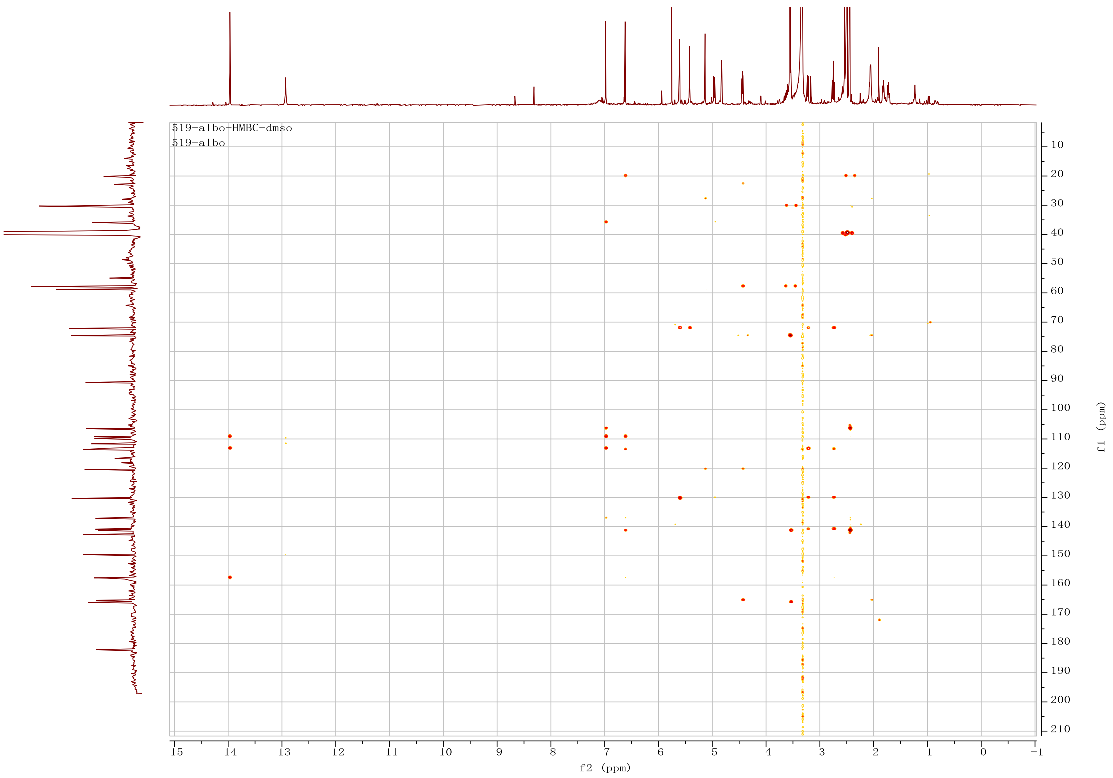

Supplementary Figure 9. HMBC spectrum of albofungin B (**2**) in DMSO-d_6.

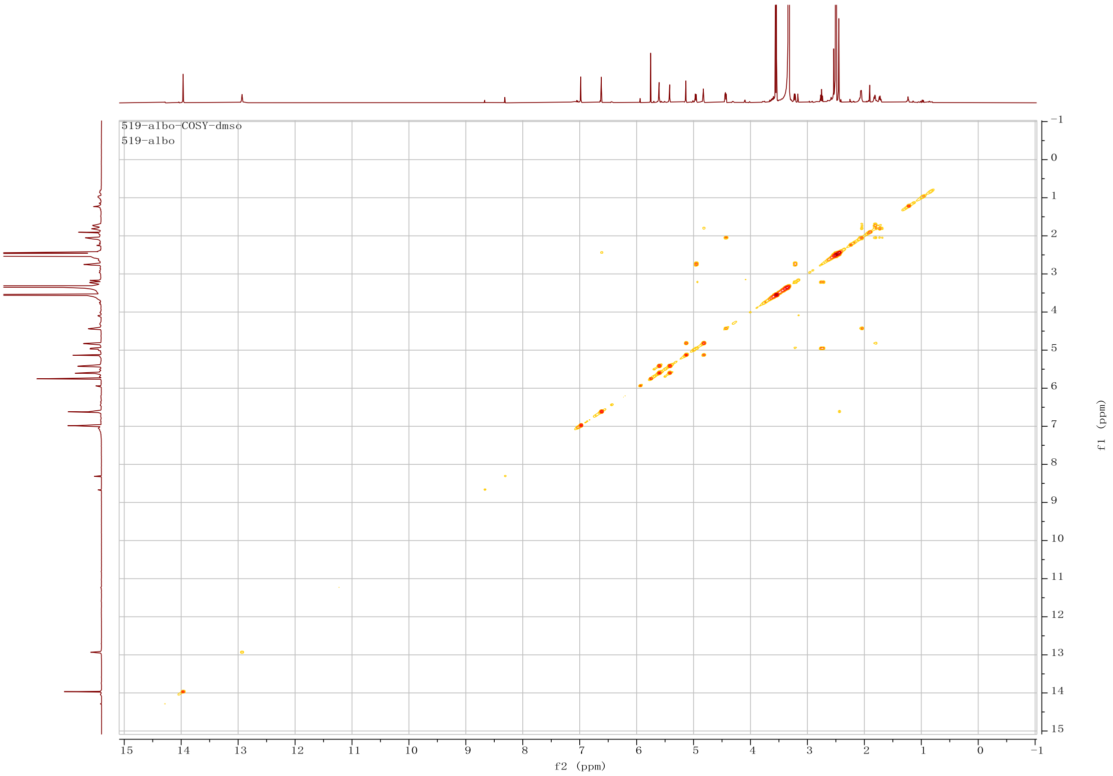

Supplementary Figure 10. COSY spectrum of albofungin B (**2**) in DMSO-d_6.

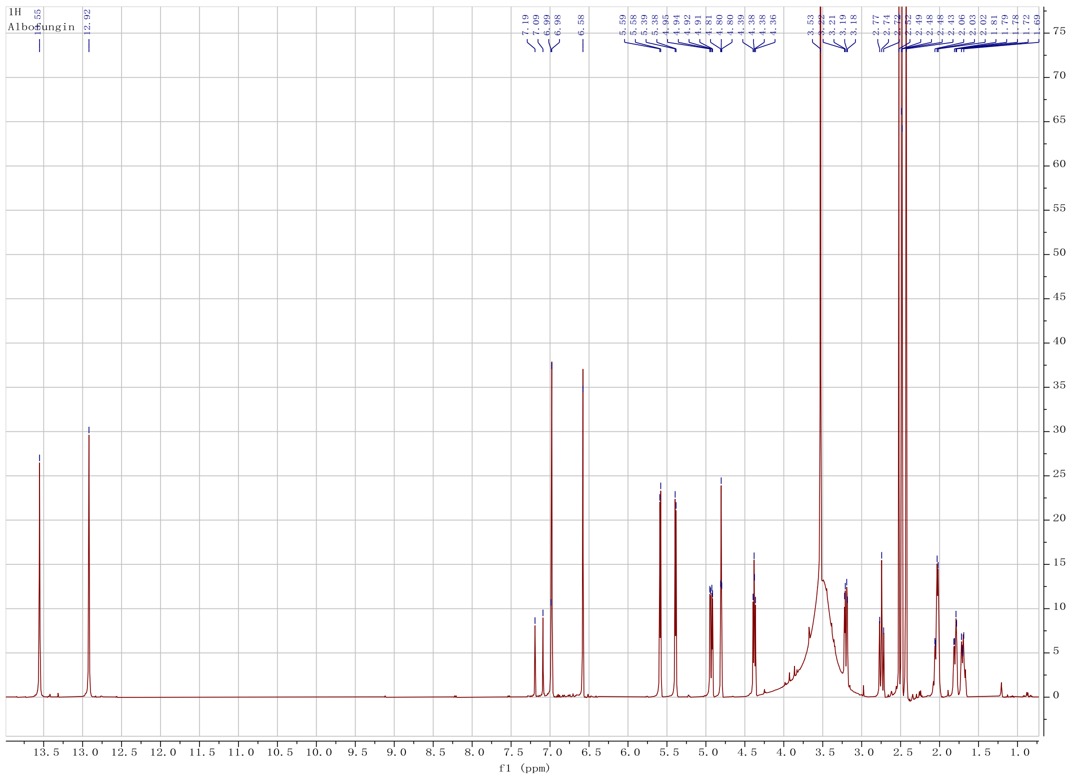

Supplementary Figure 11. ^{1}H NMR spectrum of albofungin (**3**) in DMSO-d_6.

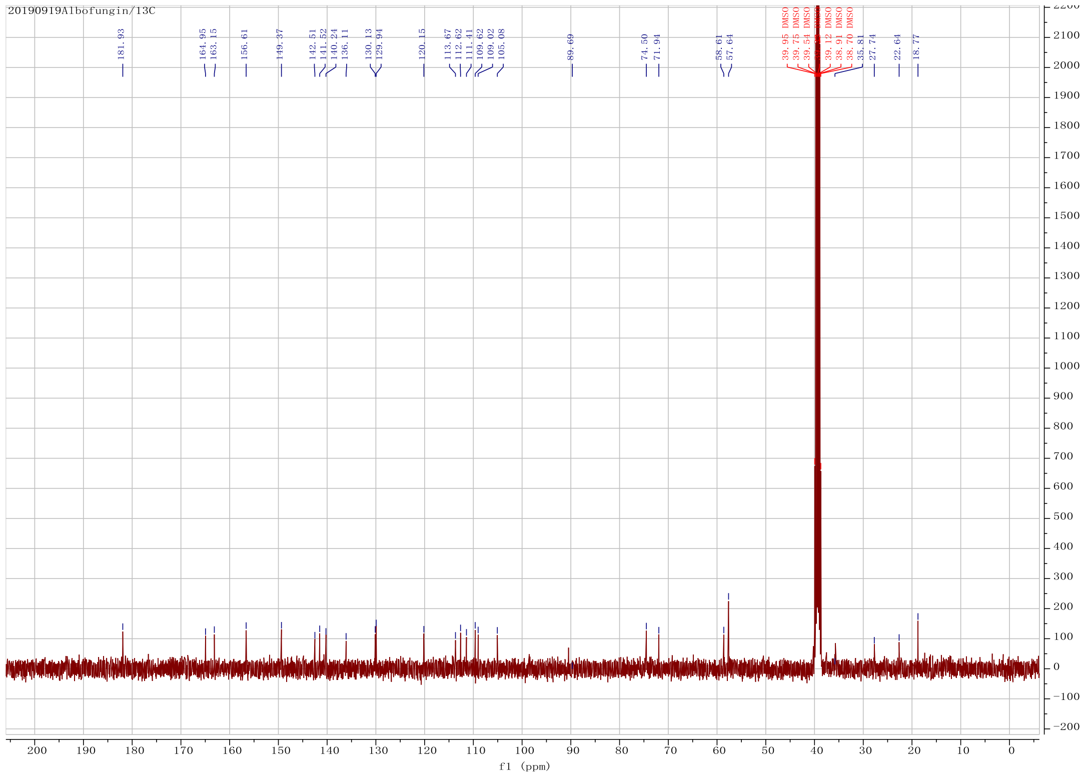

Supplementary Figure 12. ^{13}C NMR spectrum of albofungin (**3**) in DMSO-d_6.

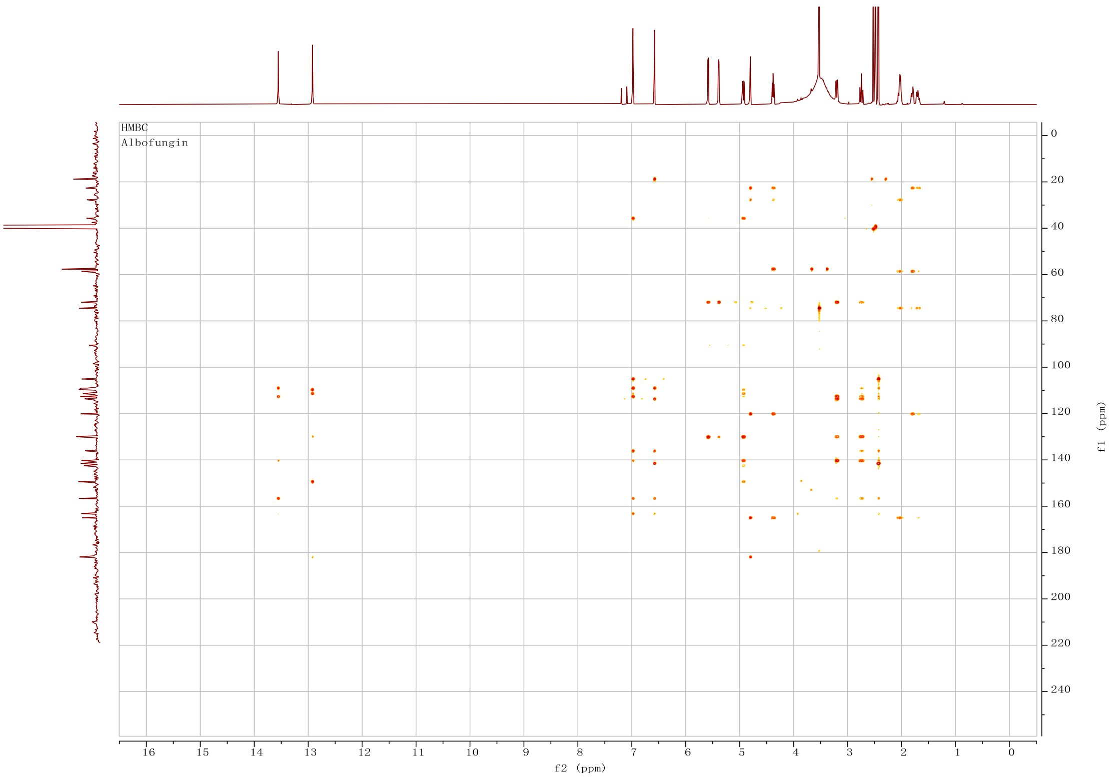

Supplementary Figure 13. HMBC spectrum of albofungin (**3**) in DMSO-d_6.

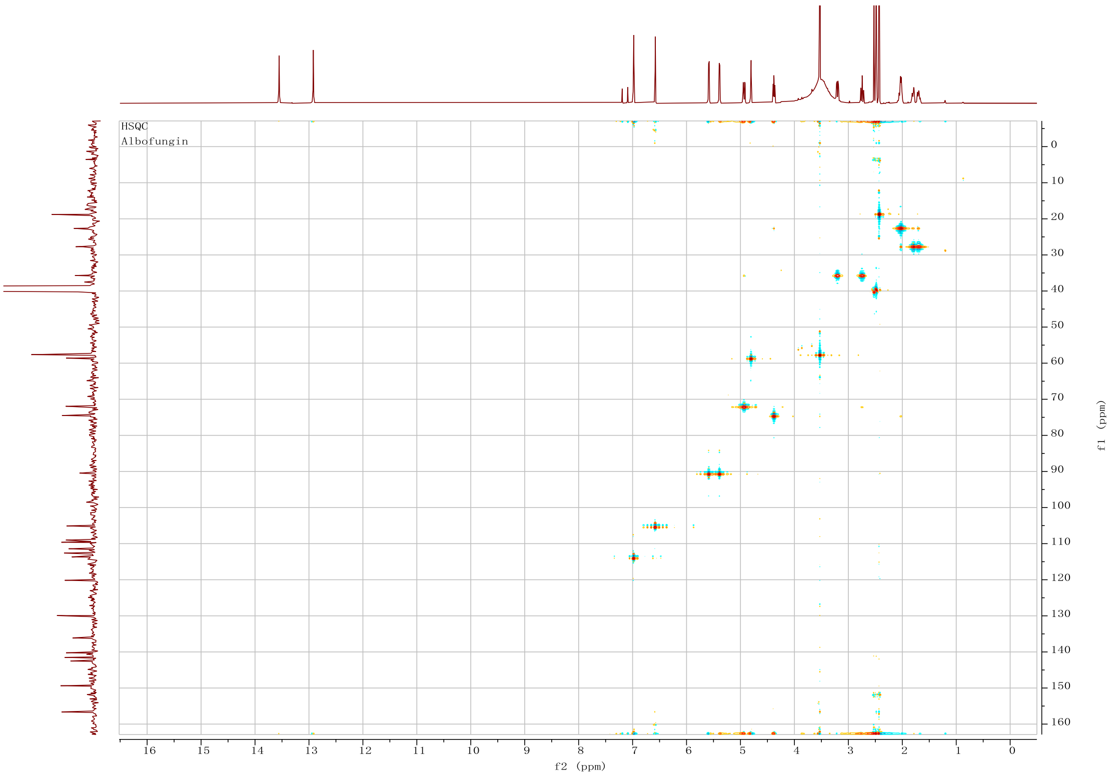

Supplementary Figure 14. HSQC spectrum of albofungin (**3**) in DMSO-d_6.

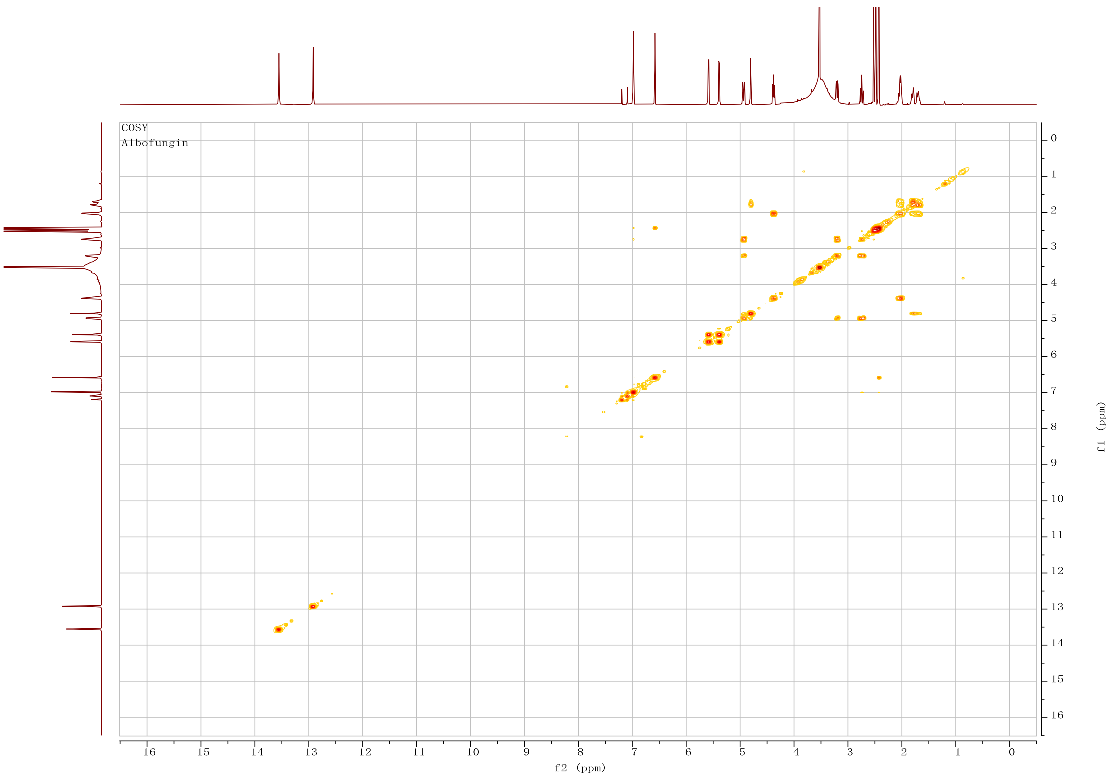

Supplementary Figure 15. COSY spectrum of albofungin (**3**) in DMSO-d_6.

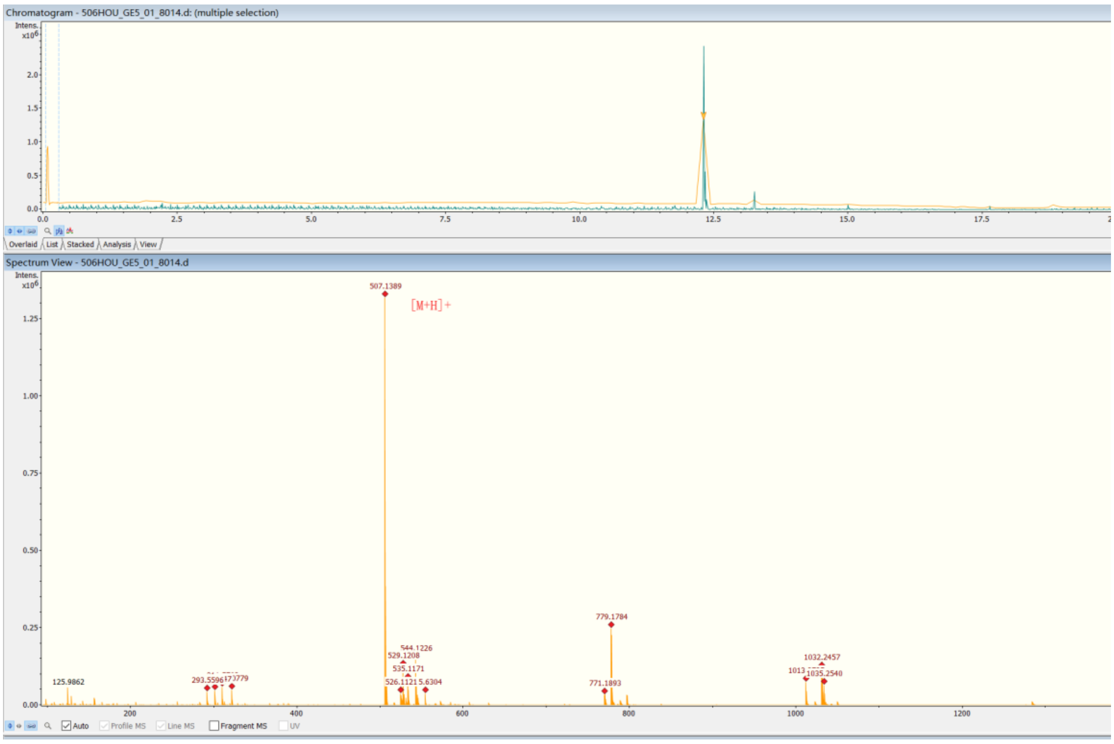

Supplementary Figure 16. HRESIMS spectrum of albofungin A (**1**).

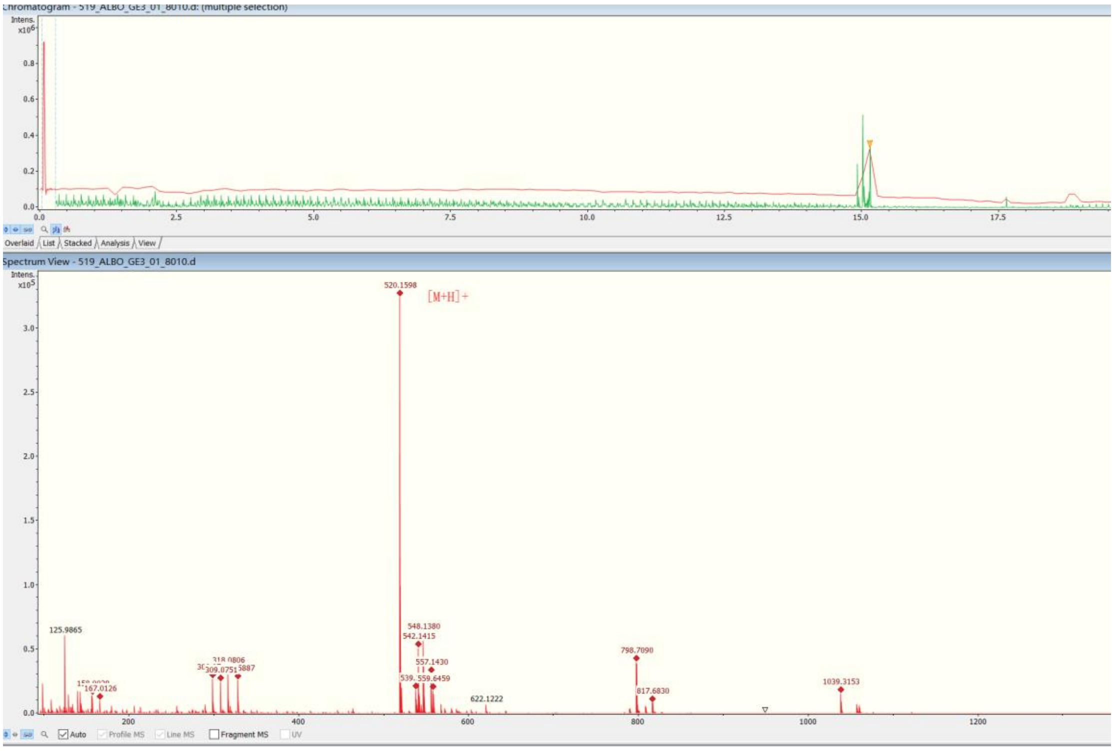

Supplementary Figure 17. HRESIMS spectrum of albofungin B (**2**).

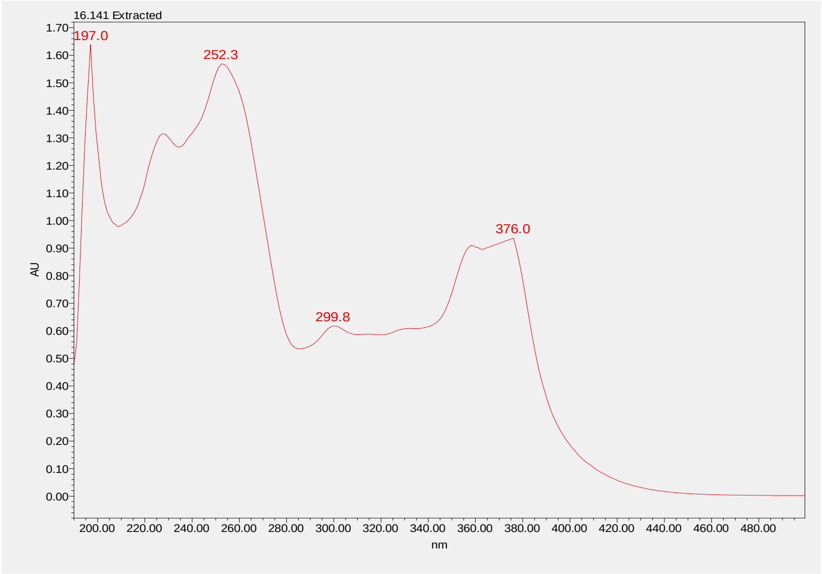

Supplementary Figure 18. UV-visible spectrum of albofungin A (**1**).

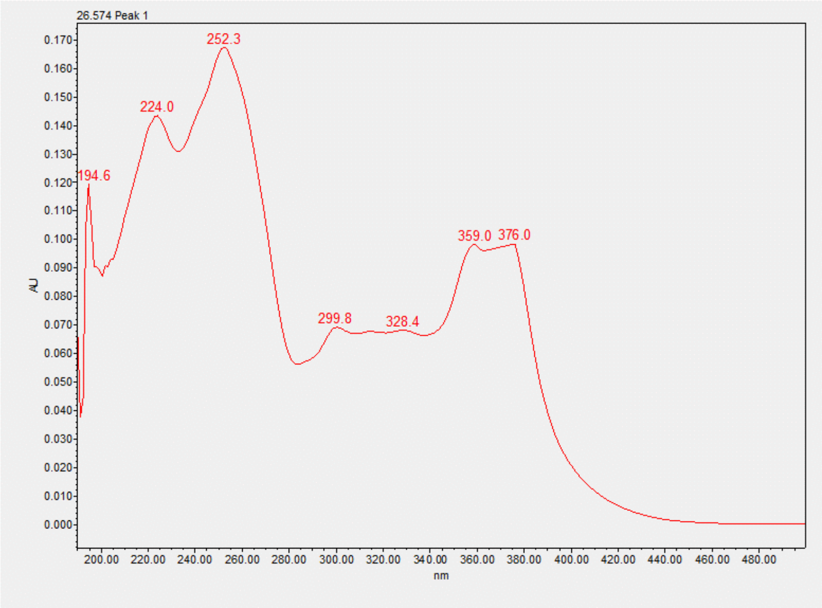

Supplementary Figure 19. UV-visible spectrum of albofungin B (**2**).

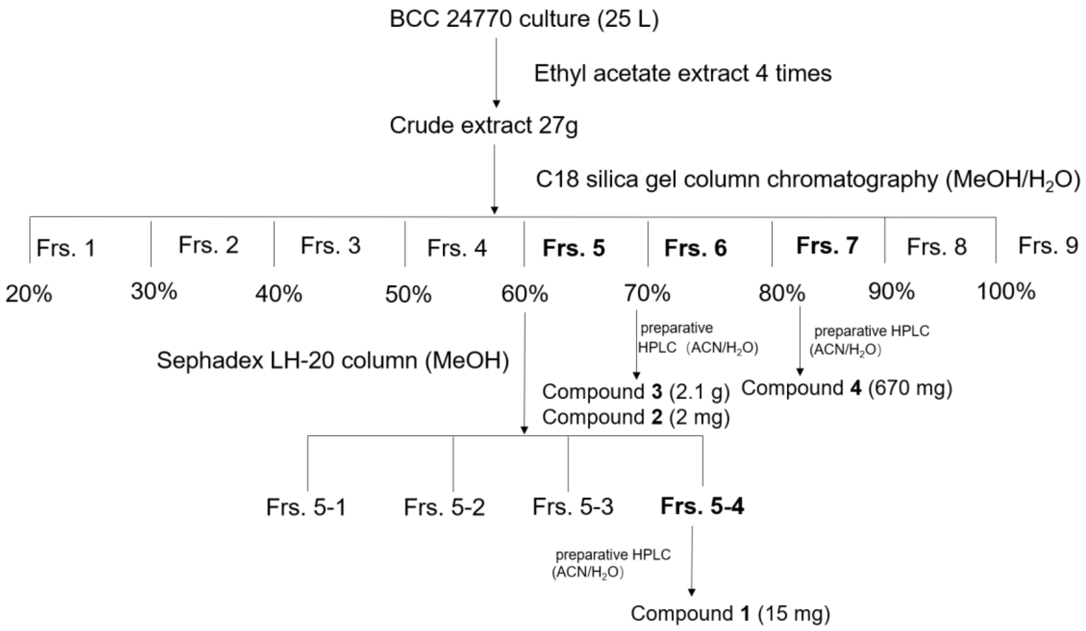

Supplementary Figure 20. The flow chart of isolation and purification of albofungin derivatives (**1-4**).

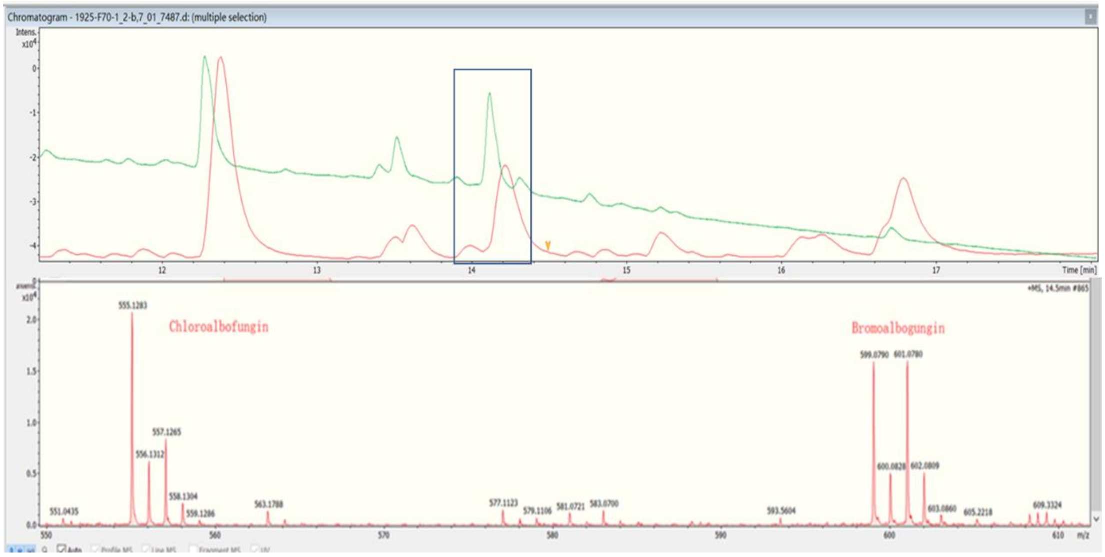

Supplementary Figure 21. UPLC-MS analysis of the bromoalbofungin from *Streptomyces chrestomyceticus* BCC 24770 .

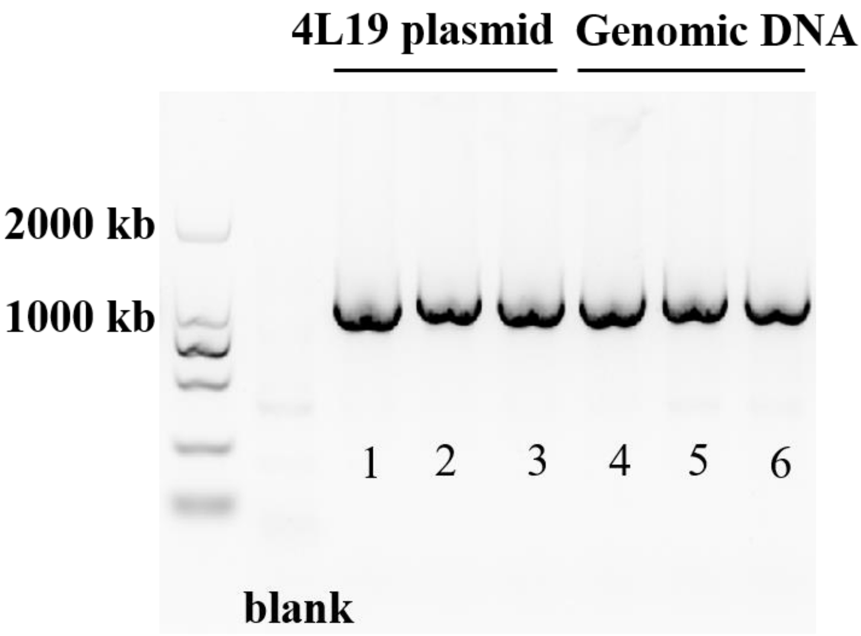

Supplementary Figure 22. BAC screening for the albofungin biosynthetic gene cluster from *Streptomyces chrestomyceticus* BCC 24770 BAC library. PCR verification of the 4L19-positive BAC plasmid. 1 and 4 are PCR products amplified using Lib-screen-up F/R, 2 and 5 are PCR products amplified using Lib-screen-middle F/R, and 3 and 6 are PCR products amplified using Lib-screen-down F/R.

A

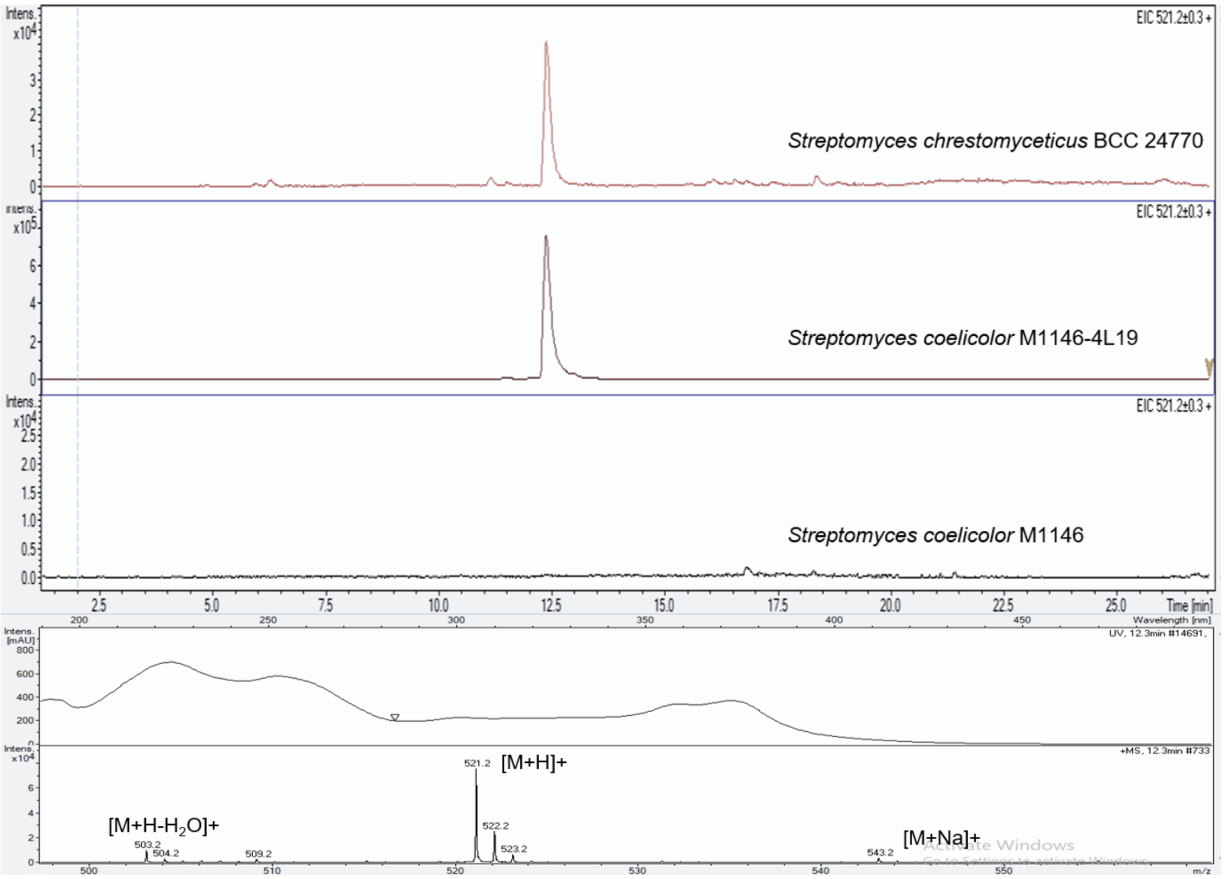
EIC 521.2±0.3 +
Streptomyces chrestomyceticus BCC 24770
Streptomyces coelicolor M1146-4L19
Streptomyces coelicolor M1146
Intens. x10^4
Intens. x10^5
Time [min]
Wavelength [nm]
UV, 12.3min #14691,
Intens. [mAU]
+MS, 12.3min #733
[M+H]+
[M+H-H2O]+
[M+Na]+
521.2
522.2
523.2
503.2
504.2
509.2
543.2
m/z

B

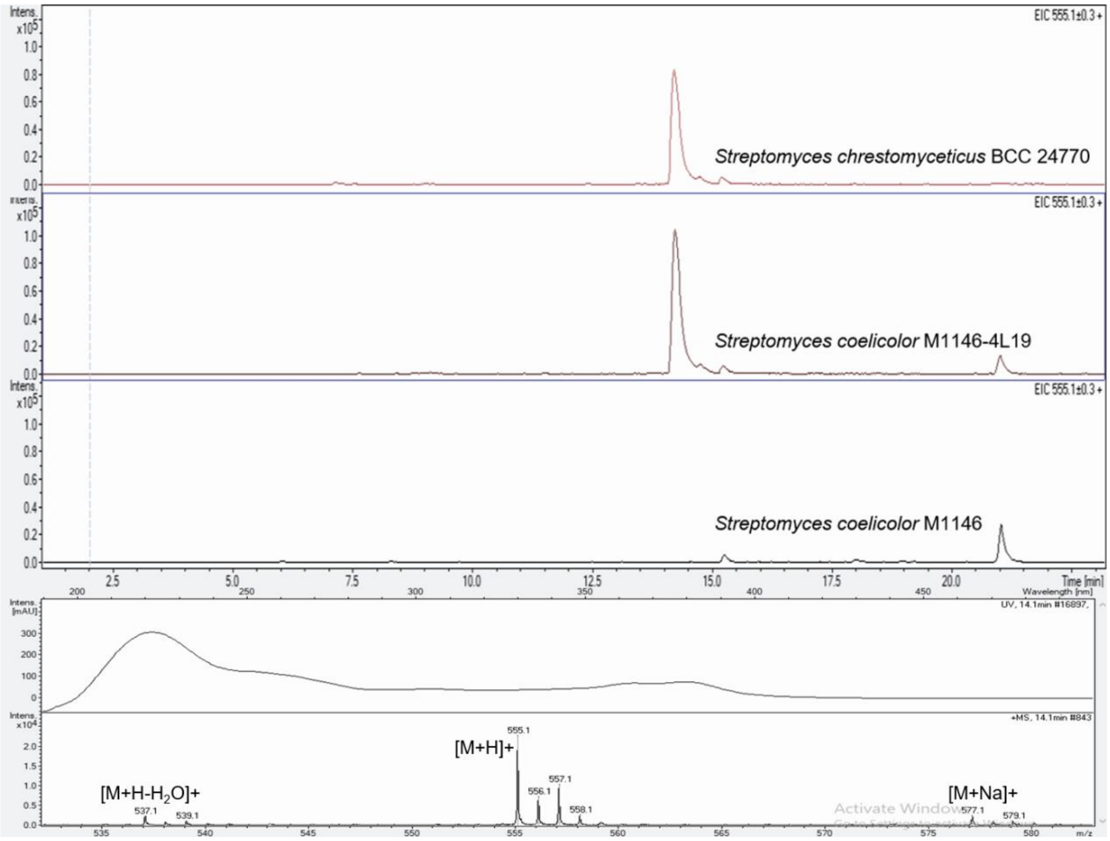
EIC 555.1±0.3 +
Streptomyces chrestomyceticus BCC 24770
Streptomyces coelicolor M1146-4L19
Streptomyces coelicolor M1146
Intens. x10^5
Time [min]
Wavelength [nm]
UV, 14.1min #16897,
Intens. [mAU]
+MS, 14.1min #843
[M+H]+
[M+H-H2O]+
[M+Na]+
555.1
556.1
557.1
558.1
537.1
539.1
577.1
579.1
m/z

Supplementary Figure 23. UPLC-MS analysis of metabolites from heterologous expression of albofungin gene cluster in *Streptomyces coelicolor* M1146. (A) Extracted ion chromatograms of albofungin (**3**). (B) Extracted ion chromatograms of chloroalbofungin (**4**).
